# Nucleic Acid-Based COVID-19 Therapy Targeting Cytokine Storms: Strategies to Quell the Storm

**DOI:** 10.3390/jpm12030386

**Published:** 2022-03-03

**Authors:** Mai Abdel Haleem Abusalah, Moad Khalifa, Mohammad A. I. Al-Hatamleh, Mu’taman Jarrar, Rohimah Mohamud, Yean Yean Chan

**Affiliations:** 1 Department of Medical Microbiology and Parasitology, School of Medical Sciences, Universiti Sains Malaysia, Kota Bharu 16150, Kelantan, Malaysia; maiabdelhaleem@student.usm.my; 2 School of Health Sciences, Health Campus, Universiti Sains Malaysia, Kubang Kerian, Kota Bharu 16150, Kelantan, Malaysia; completeuses@gmail.com; 3 Department of Immunology, School of Medical Sciences, Universiti Sains Malaysia, Kota Bharu 16150, Kelantan, Malaysia; alhatamleh@student.usm.my (M.A.I.A.-H.); rohimahm@usm.my (R.M.); 4 College of Medicine, Imam Abdulrahman Bin Faisal University, Dammam 34212, Saudi Arabia; mkjarrar@iau.edu.sa; 5 Medical Education Department, King Fahd Hospital of the University, Al-Khobar 34445, Saudi Arabia

**Keywords:** COVID-19, cytokine storm, TNA, miRNA, siRNA, mRNA vaccine

## Abstract

Coronavirus disease 2019 (COVID-19) has shaken the world and triggered drastic changes in our lifestyle to control it. Despite the non-typical efforts, COVID-19 still thrives and plagues humanity worldwide. The unparalleled degree of infection has been met with an exceptional degree of research to counteract it. Many drugs and therapeutic technologies have been repurposed and discovered, but no groundbreaking antiviral agent has been introduced yet to eradicate COVID-19 and restore normalcy. As lethality is directly correlated with the severity of disease, hospitalized severe cases are of the greatest importance to reduce, especially the cytokine storm phenomenon. This severe inflammatory phenomenon characterized by elevated levels of inflammatory mediators can be targeted to relieve symptoms and save the infected patients. One of the promising therapeutic strategies to combat COVID-19 is nucleic acid-based therapeutic approaches, including microRNAs (miRNAs). This work is an up-to-date review aimed to comprehensively discuss the current nucleic acid-based therapeutics against COVID-19 and their mechanisms of action, taking into consideration the emerging SARS-CoV-2 variants of concern, as well as providing potential future directions. miRNAs can be used to run interference with the expression of viral proteins, while endogenous miRNAs can be targeted as well, offering a versatile platform to control SARS-CoV-2 infection. By targeting these miRNAs, the COVID-19-induced cytokine storm can be suppressed. Therefore, nucleic acid-based therapeutics (miRNAs included) have a latent ability to break the COVID-19 infection in general and quell the cytokine storm in particular.

## 1. Introduction

The rapid spread of the novel coronavirus disease 2019 (COVID-19) has hit every corner of the world and has led to drastic changes in the lifestyle of humanity. It was reported that this disease is caused by the infection of severe acute respiratory syndrome coronavirus 2 (SARS-CoV-2) [1,2,3]. After that, COVID-19 was declared a global pandemic by the World Health Organization (WHO) on 30 January 2020 [2]. SARS-CoV-2 is a single-stranded RNA (ssRNA) coronavirus from a subfamily of the *Coronaviridae* family in the order *Nidovirales* [4], which has four genera: *Alphacoronavirus*, *Betacoronavirus*, *Gammacoronavirus*, and *Deltacoronavirus* [5]. The order *Nidovirales* includes enveloped positive ssRNA viruses. These viruses have caused multiple epidemics, beginning in 2002 with SARS-CoV, which was first detected in China [5], and then in 2012 with Middle East respiratory syndrome (MERS-CoV), which was first detected in Saudi Arabia [6,7].

**Figure 1:**
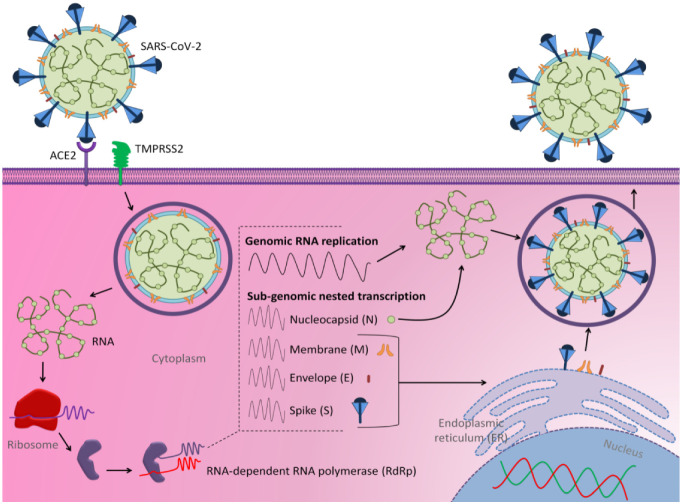
Structure scheme of SARS-CoV-2 and its mechanisms of cell entry and replication (adapted from Al-Hatamleh et al., 2020 [2]).

SARS-CoV-2 is considered part of the B lineage of *Betacoronavirus* (β-CoVs), based on sequence analysis [8]. Generally, the size of coronaviruses ranges between 65 and 125 nm in diameter and their ssRNA size from 26 to 32 kbs in length [9]. The enveloped positive-sense single-stranded RNA of the virus encodes multiple proteins, including the spike (S), membrane (M), envelope (E), and nucleocapsid (N)—the main structural proteins responsible for structural maintenance and virulence of the virus (Figure 1) [10,11]. The virus attaches to the host cell via binding of the S protein to the respective receptor via the receptor-binding domains (RBD) in the S1 region [12]. The main receptor that mediates the binding of the S protein was identified as the angiotensin-converting enzyme 2 (ACE2) [13,14], which acts as the target for impeding the commencement of the infection [15]. The viral genome gains entry via the cleavage of the S protein and fusion with the cellular membrane of the host cell [16]. Transmembrane serine protease 2 (TMPRSS2), the vital protein for cleavage, also acts as a target for blocking the entry of the virus [17,18]. The subsequent RNA replication occurs through discontinuous transcription [19] followed by translation of mainly the structural proteins that migrate to the endoplasmic reticulum–Golgi intermediate compartment (ERGIC) [20], in which the budding of the N encapsidated viral genome takes place to form mature virions [21]. The virions are then typically transported by vesicles to be excreted (Figure 1).

Similar to many viruses, the detection of SARS-CoV-2, especially from nasopharyngeal swab samples [22,23,24], is done by using reverse transcription polymerase chain reaction (RT-PCR) techniques, which are considered the gold standard tests [11,25]. On the other hand, during the early months of the pandemic, many drugs have been investigated as potential COVID-19 treatments, including repurposed or experimental drugs [26,27]. However, no efficient drug has been found so far, including the Food and Drug Administration (FDA)-approved ones for emergency use, such as remdesivir, which showed a decreased median recovery time of 10 days instead of 15 days for the placebo patients [28]. Hence, no decisive antiviral treatment has been investigated. The world believes that the invention of COVID-19 vaccines is the ideal way to quell this global pandemic and restore a normal lifestyle, where antiviral therapeutics could play a role as supportive treatment options.

The astonishing number of confirmed cases and deaths globally has spurred the rapid development of therapeutics and vaccine approaches. The urgent need for effective vaccines and antiviral therapeutics has made it urgent to explore promising unconventional technologies that have the advantage of rapid development towards the swift counteraction of the pandemic [29]. Therefore, in the early months of the COVID-19 pandemic, several research groups and institutions had realized that the potential solution could lie within the nucleic acid-based approaches, which allow for the quick response needed to overcome this global pandemic. Thus, several nucleic acid-based therapeutics and vaccines have been proposed. Two of them (i.e., the Moderna and Pfizer–BioNTech vaccines) are among six vaccines that passed the clinical evaluations and received emergency authorization [30]. Although nucleic acid-based technology is a relatively new class for developing therapeutics and vaccines, it has surprised the scientific community and shown effective and promising outcomes. Therefore, the current revolution in nucleic acid-based technology to develop therapeutics and vaccines against COVID-19 deserves to be discussed in depth. This review summarizes the recent advances in developing nucleic acid-based therapeutics and vaccines against COVID-19 and suggests a potential nucleic acid-based therapy for targeting cytokine storms in COVID-19 patients.

## 2. Current Nucleic Acid-Based Approaches against COVID-19

In the field of next-generation vaccines, nucleic acid approaches have attracted a lot of attention. The first proof of concept for a DNA vaccine was performed in 1990, and it involved injecting RNA or DNA molecules, expressing luciferase, beta-galactosidase, and chloramphenicol acetyltransferase, into mouse skeletal muscle, and expressing reporter genes in vivo, which can be found for up to two months after infection [31]. Since 1990, many institutions have begun working on plasmid DNA and messenger RNA (mRNA) for vaccines, cancer immunotherapies, and immunological treatments for autoimmune and allergy disorders [32]. Over the last few years, a lot of effort has gone into the research and production of DNA or RNA vaccines [32]. Improvements in the DNA and RNA vaccine development methods are critical, given the rising frequency of epidemics. Furthermore, efficient vaccine development may avoid infections caused by highly transmittable viruses. Because synthetic DNA and RNA are easier to create, a DNA and RNA-based method allow for more rapid vaccine development [33].

Although the technology of nucleic acid-based vaccines is being used for the first time, it shows very interesting outcomes. The nucleic acid-based vaccination methods employ RNA (mRNA) [34] or plasmid DNA, both of which encode antigens [33]. When these antigens are expressed following cellular uptake, they can elicit both humoral and cell-mediated immune responses [35]. Because it allows for simple maneuvering and modification of the antigen, the nucleic acid-based vaccination technique is considered versatile and adaptable [33]. Of the 36 RNA-based COVID-19 vaccine candidates, 22 are still under the pre-clinical stage, while 12 passed to the clinical stages and two were authorized (Table S1) [36,37,38,39,40,41,42,43,44,45,46,47,48,49,50,51,52,53,54,55,56,57,58,59,60,61,62,63,64,65]. The Pfizer–BioNTech vaccine was the first approved COVID-19 vaccine worldwide, which is the most desirable vaccine and found to be effective against variants (i.e., the B.1.1.7, B.1.351, and B.1.617.2 variants) [66,67,68]. The only vaccines that have been authorized for emergency use by the FDA are the Moderna and Pfizer–BioNTech vaccines, which have been made using mRNA technology, in addition to the Janssen vaccine [69].

Under an emergency-use authorization, the FDA has approved the use of the Pfizer–BioNTech COVID-19 vaccine to prevent COVID-19 in people older than 12 years of age [70]. The Pfizer–BioNTech (BNT162b2) vaccine is a lipid nanoparticle-formulated, nucleoside-modified RNA (modRNA) encoding the SARS-CoV-2 full-length S protein, which has been modified by two proline mutations to lock it in the prefusion conformation [41,42,71,72,73].

The following components are included in the Pfizer–BioNTech COVID-19 vaccine: mRNA; lipids ((4-hydroxybutyl) azanediyl) bis (hexane-6,1-diyl) bis (2-hexyldecanoate), 2 [(polyethyleneglycol)-2000]-N, N-ditetradecylacetamide, 1, 2-Destearoyl-sn-glycero-3-phosphocholine, and cholesterol), where the primary function of the lipids is protecting the mRNA and creating a greasy coating that allows the mRNA to enter the cells; salts (monobasic potassium phosphate, potassium chloride, dibasic sodium phosphate dihydrate, sodium chloride), which helps in balancing the acidity inside the human body; and sucrose (helps in the preservation of the form of the molecules during freezing). The Pfizer–BioNTech COVID-19 vaccine is administered by muscle injection [70,74]. The Pfizer–BioNTech COVID-19 vaccine immunization series consists of two doses administered three weeks apart [70].

Unlike vaccinations that introduce weakened or inactivated disease viruses into the body, the Pfizer mRNA vaccine presents a small piece of genetic code from the SARS-CoV-2 virus to the host cells [71]. Following injection, the vaccine’s mRNA is released into the cells’ cytoplasm [74]. Once the viral protein is created on the cell’s surface, the mRNA is broken down and eliminated by the body, making it unable to modify our DNA [74]. The mRNA vaccine’s genetic code effectively gives host cells instructions, or blueprints, for manufacturing copies of S proteins. The S proteins are responsible for entering and infecting host cells [71]. These proteins elicit an immune response, resulting in the production of neutralizing antibodies and eliciting robust interferon-γ (IFN-γ)-producing and interleukin-2 (IL-2)-producing CD8+ cytotoxic T-cell and CD4+ type 1 helper T (Th1) cell responses that will identify the virus and respond consequently if the body becomes infected with the real virus [41,75]. In April 2021, the company revealed that the vaccine showed 91.3% effectiveness against COVID-19, based on how effectively it prevented symptomatic COVID-19 infection seven days after the second dosage for up to six months [51]. Moreover, in early May 2021, the Pfizer vaccine was proven to be more than 97.4% effective against severe disease or death from the Alpha variant (initially discovered in the United Kingdom) and the Beta variant (first identified in South Africa) [67,76,77,78]. Concerning the Delta variant (first observed in India), non-peer reviewed studies published by Public Health England found that complete vaccination after two doses is 88% effective against symptomatic illness and 96% effective against hospitalization [76,77,79].

A week after the Pfizer vaccine authorization, Moderna’s COVID-19 vaccine (mRNA-1273) was also approved for emergency use in the United States [71,80,81]. The Moderna vaccine is a lipid nanoparticle-encapsulated mRNA vaccine that expresses the prefusion-stabilized S glycoprotein [82,83]. The Moderna vaccine has high effectiveness in symptomatic disease prevention, similar to the Pfizer vaccine. In addition, the Moderna vaccine has a similar mechanism of action as the Pfizer vaccine [71,80,81]. The Moderna COVID-19 vaccine includes the following ingredients: mRNA; sucrose and salts (sodium acetate trihydrate) to maintain the stability of the vaccine; lipids (SM-102, polyethylene glycol [PEG] 2000 dimyristoyl glycerol [DMG], cholesterol, and 1,2-distearoyl-sn-glycero-3-phosphocholine [DSPC]) to help in delivering the mRNA to the cells; and acids (acetic acid) and acid stabilizers (tromethamine hydrochloride) to maintain the stability of the vaccine [74,84]. The Moderna COVID-19 vaccine is administered as an injection into the muscle, with two doses administered one month apart [84]. In June 2021, Moderna announced that the Moderna vaccine is effective against the Beta, Delta, Eta, and Kappa variants; however, it was found to be roughly two times weaker against Delta than against the original virus [83,85,86], indicating that additional studies on Moderna’s efficiency against the Delta variant is required.

Moreover, there are six potential RNA-based treatments being developed to combat COVID-19 infection, but none of them has been authorized so far (Table S2) [87,88,89,90]. Interestingly, the preclinical evaluation for two of them (i.e., OT-101 and Ampligen) showed promising results; thus, they had successfully moved to the clinical trials stage.

On 6 April 2020, Mateon Therapeutics Inc. committed to developing OT-101, a transforming growth factor beta (TGF-β) antisense medicine candidate, and stated that this treatment could be utilized to treat COVID-19 [91]. TGF-β is involved in various biological processes, including embryogenesis, tissue regeneration, immunological responses, and cancer. TGF-β may potentially function as a pro-viral factor. TGF-β upregulation is also implicated in the pathogenesis of several respiratory diseases, including pulmonary fibrosis, emphysema, bronchial asthma, and lung cancer. Boumaza et al. demonstrated that SARS-CoV-2 effectively infects monocytes and macrophages in vitro, resulting in TGF-β secretion [92]. Furthermore, SARS-CoV-2 infection decreases the expression of ACE2, which causes local vascular inflammation and stimulates TGF-β production via aldosterone. TGF-β controls various cellular processes, including those involved in the development of acute lung injury (ALI)/acute respiratory distress syndrome (ARDS). Patients with ARDS who had a lower TGF-β level in their bronchoalveolar lavage fluid (BALF) had fewer possibilities to get mechanical ventilation and spent less time in the intensive care unit (ICU) [93]. Therefore, OT-101 was designed to block TGF-β, which might be very useful for COVID-19 patients. OT-101’s mechanisms of action against COVID-19 are as following: (1) reduce TGF-β production/secretion [94]; (2) inhibition of cellular binding; (3) inhibition of viral replication; and (4) suppression of viral-induced pneumonia [91,95]. Mateon thinks that OT-101 will be beneficial for future viral outbreaks based on its mechanism of action. With a high safety index of >500, OT-101 demonstrated significant efficacy against both COVID-19 and SARS [91]. Furthermore, Mateon has submitted a Pre-Investigational New Drug (Pre-IND) application package to the FDA to allow the referencing of OT-101’s oncology IND to streamline OT-101’s IND submission against COVID-19 [91].

One of the most effective natural defense mechanisms against viral infection is IFN release. In vivo studies revealed that interferon beta-1b (IFN-β1b) therapy decreased the lung infiltrates, bronchointerstitial pneumonia, and viral load of MERS-CoV [96]. IFN-λ has also shown antiviral efficacy against SARS-CoV and MERS-CoV, producing an antiviral state while causing low systemic inflammation [97]. In 196 studies, antibodies against cytokines and other proteins were provided, whereas 58 studies focus on IFN, either by suppressing them or administering their recombinant form to treat patients [98]. Rintatolimod (Ampligen) is the only Toll-like receptor-3 (TLR3) agonist (immune adjuvant that potently stimulates innate immune response) that promotes selective recruitment of CTLs (cytotoxic T lymphocytes) with a concurrent rise in intratumoral effector T cell (Teff)/intratumoral regulatory T cell (Treg) ratio in the tumor microenvironment [99]. Following the SARS-CoV epidemic in 2003, Ampligen showed strong antiviral properties and a protective survival impact in National Institutes of Health (NIH)-contracted trials of SARS-infected mice, which is nearly identical to SARS-CoV-2. According to the Barnard 2006 study, the only drugs that effectively reduced viral titers in the lungs of infected mice were hybrid interferon, IFN-α B/D, and a mismatched double-stranded RNA (dsRNA) IFN inducer, Ampligen® (poly I: poly C124), where Ampligen decreased viral lung levels to undetectable levels [100]. Furthermore, the Day 2009 study discovered that, instead of 100% death, there was 100% protective survival [101]. AIM compared key transcription regulatory sequences from SARS-CoV-1 to SARS-CoV-2 and discovered strong and convincing similarities, suggesting that the antiviral benefits of Ampligen in earlier NIH-contracted SARS studies might most likely extend to COVID-19 [99]. This makes a strong case for clinical trials to evaluate Ampligen as a potential tool in the battle against COVID-19.

Ampligen is a known inducer of IFN, a synthetic dsRNA polymer that stimulates interferon production, and has been demonstrated in vivo to be effective against a variety of viruses [100,101]. Ampligen at 10 mg/kg was previously reported to protect against SARS-CoV replication in the lungs in a viral replication model. IFNs are usually produced and released by infected cells in the presence of double-stranded RNA or 5′-triphosphorylated ssRNA, as in viral infection, and promote the production of potent antiviral proteins [102]. However, IFN pathways are inhibited in SARS-CoV-infected cells, rendering the host more susceptible to infection. Exogenous activation of IFN pathways using Ampligen overcomes the evasion of viral sensing and signaling pathways [103]. Ampligen did not appear to influence viral replication at first, but it did result in a faster drop of virus in the lungs when compared to untreated animals. A prior study found that Ampligen protected against mortality and severe lung damage in the presence of the deadly SARS-CoV [101].

There is growing anecdotal evidence that people recovering from COVID-19 can acquire a Myalgic Encephalomyelitis/Chronic Fatigue Syndrome (ME/CFS)-like condition known as COVID survivor syndrome or COVID-19-induced chronic fatigue syndrome [104]. Individuals suffering from COVID-19 have been known as long-haulers. This post-viral syndrome, which includes brain fog, tiredness, and trouble concentrating, has been reported to continue for many weeks following COVID-19 clinical recovery and SARS-CoV-2 clearance. Ampligon is being investigated as a possible treatment for COVID-19-induced chronic fatigue syndrome [104].

On the other hand, several DNA-based vaccine candidates have been proposed and are being tested against COVID-19 (Table S3) [105,106,107]. Although none of the DNA-based COVID-19 vaccine candidates is authorized so far, 12 of them are currently under clinical evaluation. ZyCoV-D, AG0302/AG0301-COVID, and INO-4800 are promising DNA-based COVID-19 vaccine candidates that reached Phase II/III and III clinical trials.

The Indian company Cadila Healthcare Ltd. developed the ZYCOV-D vaccine candidate [108]. This vaccine is new plasmid DNA vaccine technology, where it was created utilizing a DNA vaccine platform that included a non-replicating and non-integrating plasmid containing the desired gene [109]. The pathogen’s antigen encoding sequence is engineered into recombinant plasmid DNA [110]. When plasmid DNA is injected into host cells and the viral protein is translated, it triggers a powerful immunological response that stimulates the immune system’s humoral and cellular components [109]. This DNA vaccine platform has minimal biosafety requirements (BSL-1), better stability, and reduced cold-chain needs [109]. ZyCoV-D is a three-dose intradermal vaccination that can significantly reduce adverse effects [111,112]. Furthermore, the ZyCoV-D platform can be modified easily in a few weeks if the virus mutates, ensuring that the vaccination continues to provide protection [109]. Phase I clinical trials of this vaccine candidate were conducted in July 2020, where the company reported adequate dosing and tolerance [108,113]. Followed by Phase II clinical trials, which were conducted in August 2020 [108]. The ZyCoV-D vaccine has been granted permission by the Drugs Controller General of India (DCGI) to conduct a Phase III clinical trial, where Phases I, II and III were monitored by an independent data safety monitoring board [114]. According to the company, because ZyCoV-D is a plasmid DNA vaccine, it has no problems with vector-based immunity [111]. In addition, the company has evaluated the difference in immunogenicity between the two-dose regimen and three-dose regimen, and equivalent immunogenicity was reported [114].

The Japanese company AnGes., in cooperation with Osaka University, recently completed Phase I/II studies of the AG0301 COVID-19 and AG0302 COVID-19 vaccines [115]. These vaccines are DNA plasmid vaccines which been given with adjuvant [116]. AnGes, Inc.’s vaccine disrupts the link between the coronavirus’s S protein and the receptors on human cells. AnGes, Inc.’s vaccine candidate encodes the SARS-CoV-2 S protein, which opens the door to infection. The vaccine generates antibodies by expressing only the S proteins within the body [117]. Then, the antibodies are produced in response to the innocuous tips, resulting in resistance. Antibodies attach to the viral S protein when it enters the body, preventing the virus from binding to the cell’s receptor. Therefore, antibodies trap the virus before it reaches the cells. It is designed to keep COVID-19 patients’ conditions from worsening. The vaccine is nonpathogenic and safe because it is designed to express just the S proteins on the viral surface, resulting in antibody production [117]. Following Phase I/II clinical trials, AnGes announced cooperation with Colorado-based Brickell Biotech on 8 September 2020 [118]. On 7 December 2020, a Phase II/III randomized, double-blind, placebo-controlled study to assess efficacy, immunogenicity, and safety of twice dosing of intramuscular AG0302 COVID-19 (2 mg) in healthy adults was commenced [119].

The INO-4800 vaccine developed by the American pharmaceutical company Inovio, which is a DNA plasmid vaccine for SARS-CoV-2 that can be used as a prophylactic vaccine [116,120]. The plasmid pGX9501, which encodes the whole length of SARS-CoV-2’s S glycoprotein, is incorporated in this vaccine [121]. The IgE leader sequence is also attached to a codon optimized S protein sequence of SARS-CoV-2. The existence of functional antibodies and a T cell response in preclinical studies suggest that the vaccine can induce an efficient immune response within seven days following vaccination [122]. The preliminary Phase I data indicated that INO-4800 vaccine has successfully elicited neutralizing and ACE2-blocking antibodies against both the D614 and G614 SARS-CoV-2 strains in rhesus macaques [123]. In addition, 94% of participants developed an immune response to this vaccine following vaccine administration by electroporation [124]. The findings confirmed INO-4800’s immunological effect in generating both humoral and cellular adaptive immune responses, which are likely crucial for providing long-term protection against COVID-19 infection [123]. CELLECTRA, which is an Inovio-developed hand-held smart device, promotes the path for activation immunotherapy. CELLECTRA help in opening tiny holes in the cell reversibly using a short electrical pulse, allowing plasmids to enter [121]. This one-of-a-kind technology delivers optimized DNA into cells, which is translated into proteins that stimulate the immune system, resulting in a robust and specific T cell and monoclonal antibody response [121]. Once the plasmids start multiplying inside the cell, the body’s natural T and B cell response mechanisms will increase, hence will prevent the S protein from binding to the human angiotensin I-converting enzyme 2 (hACE2) receptor [112,121,123]. The vaccine was well-tolerated and immunogenic in all age groups who were tested in Phases I and II clinical trials [122,123,125,126,127,128]. In preclinical models, the company announced on 12 May 2021, that INO-4802 is the next-generation Pan-COVID-19 vaccine candidate, where this vaccine induced T cell responses and neutralizing antibodies against the original Wuhan strain, as well as B.1.1.7 (UK variant), B.1.351 (South African variant), and P.1. (Brazilian variant). These findings showed that Inovio’s Pan-COVID-19 vaccination can elicit cross-reactive immune responses against both existing and emerging virus strains [129].

### Cytokine Storm

COVID-19 patients demonstrate elevated serum IgM, IgG, and IgA levels, with even higher levels in severe cases [130]. Inflammatory mediators such as IFN-γ, tumor necrosis factor (TNF), IL-2, IL-6, and IL-10 were also elevated in COVID-19 patients, and higher levels were reported in the severe cases [131,132]. In addition, IL-1β and IL-8 were reported with a high level in infected patients [133]. The increase in cytokines (hypercytokinemia) leads to a lethal inflammatory response referred to as a cytokine storm [131]. In severe cases of about 15% of COVID-19 patients, life-threatening ARDS was found due to alveolar damage by the inflammatory mediators [134]. Targeting the inflammatory response-regulating pathways, such as the Janus kinase-signal transducer and activator of transcription (JAK-STAT) pathway, might protect from ARDS development [135]. However, in critical cases, treatment or elimination of the cytokine storm might be complex, even with many involved therapeutic targets in the immunopathogenesis [136]. Therefore, early cytokine storm management has significant value in reducing mortality, especially in severe cases.

**Figure 2:**
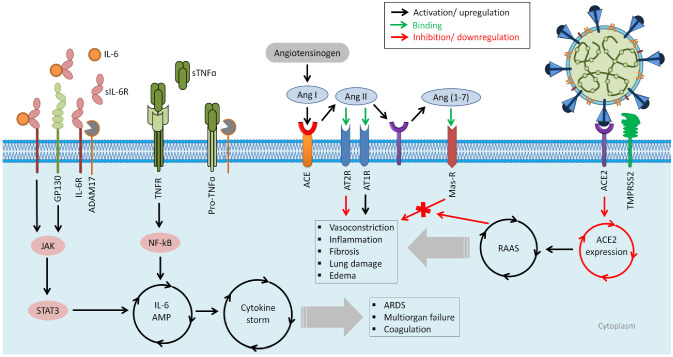
Scheme of possible targets for developing therapeutics against COVID-19 and the presented cytokine release syndrome (CRS). SARS-CoV-2 utilizes ACE2 and TMPRSS2 as cell entry receptors. As a result of SARS-CoV-2 infection, the renin–angiotensin system (RAAS) is overactive. Angiotensin-converting enzyme (ACE) converts angiotensin I (Ang I) to angiotensin II (Ang II) under physiological circumstances, resulting in increased vasoconstriction, inflammation, fibrosis, lung injury, and edema. Ang I is inactivated by angiotensin-converting enzyme 2 (ACE 2), which produces angiotensin 1–7 (Ang 1–7), which interacts with the G-protein-coupled receptor Mas. Because it antagonizes the effects of Ang I, this interaction is thought to be vasoprotective. SARS-CoV-2, on the other hand, decreases ACE2 expression, resulting in RAAS overactivation and increased lung injury and edema. Through angiotensin receptor type 1 (AT1R), AngII functions as a vasoconstrictor and a pro-inflammatory cytokine. AT1R can promote fibrosis, inflammation, acute lung injury, and collagenase activity decrease. Instead, AT2R stimulation protects against renin–angiotensin system (RAS) activation by eliciting anti-inflammatory, antioxidant, anti-fibrotic effects, reduction in vascular permeability, and edema. The hyper-activation of the NF-κB transcription factor by SARS-CoV-2 through pattern recognition receptors (PPRs) causes cytokine-related syndrome (ARDS). Angiotensin 2 (AngII) elevates in the serum because SARS-CoV-2 occupies ACE2 molecules, which, in turn, reduces Ang II breakdown. The inflammatory cytokines, including TNFα and IL-6-soluble (s)IL-6R, are activated by the accumulated AngII via disintegrin and metalloprotease 17 (ADAM17), and the IL-6 amplifier (IL-6 AMP) is subsequently activated, which explains the magnified NF-κB activation by the co-activation of NF-κB and STAT3 transcription factors. In a variety of IL-6Ra-negative nonimmune cells, including fibroblasts, endothelial cells, and epithelial cells, ADAM17 stimulation also changes the membrane form of IL-6Ra to the soluble form (sIL-6Ra), followed by the gp130-mediated activation of STAT3 via the sIL-6Ra–IL-6 complex. As a result, the simultaneous inflammatory cascades of NF-κB and STAT3-mediated signaling further enhance the NF-B activity and form an inflammatory circuit, the IL-6 AMP, which defines an IL-6-based positive feedback loop for inflammation. As a result, the cytokine storm generated by NF-B hyperactivation in IL-6 AMP might result in lethal symptoms such ARDS, severe pneumonia, multiorgan failure, and coagulation. The underlined molecules indicate possible therapeutic targets for cytokine release syndrome (CRS).

The elevated level of inflammatory mediators often correlated with the SARS-CoV-2 infection severity, including cytokines and chemokines such as IL-2, IL-6, IL-10, TNF, C-reactive protein, ferritin, and D-dimers [137]. The IL-6 blood level correlates with the SARS-CoV-2 infection mortality [138]. Therefore, the high levels of inflammatory mediators (cytokine storm) indicate that SARS-CoV-2 lethality, which is presented as cytokine release syndrome (CRS) [139], could be managed via IL-6 inhibition [140]. Figure 2 illustrates the previously discussed targets, and more, for combating the SARS-CoV-2 infection. The inflammatory mediators and other possible targets could be the aim of nucleic acid-based therapeutics to develop novel and perhaps effective therapeutics for controlling the COVID-19 infection risk.

## 3. COVID-19 Nucleic Acid-Based Therapeutics

Due to the periodical emergence of new versatile CoVs, which is considered a severe health concern [141], as is the case with SARS-CoV-2, and especially the new emerging variants with more lethality and transmissibility, the urgent development of novel efficient therapies is highly recommended. The variant of concern (VOC-202012/1) named B.1.1.7 (Alpha), originating in southeast England in 2020, was reported with high mortality rates from 2.5 to 4.1 per 1000 detected cases as compared to other circulating variants in England [142]. These variants also can escape the immune system via multiple recorded mutations, mainly E484K [143]. Delta (B.1.617.2) is presently the most prevalent COVID-19 variant in the U.S. [144]. This variant was discovered for the first time in India in December of 2020 [145]. In addition, this particular variant expanded to over 98 countries throughout the world in a matter of months, becoming the dominant variant in most countries, including India, the U.K., Israel, and the U.S. [146]. The Delta variant currently accounts for more than 83% of COVID-19 cases recorded in the U.S. [147] and it was found to be 40–60% more transmissible than Alpha, about twice as transmissible as the initial SARS-CoV-2 Wuhan strain [148]. Some monoclonal antibody therapies and antibodies produced by COVID-19 vaccination may be less successful against this variant [144]. On 16 July 2021, the Centers for Disease Control and Prevention reported a 35% rise in hospitalizations and a 69.3% seven-day average increase in new COVID-19 cases infected with a Delta variant [147].

New variants are continually developing, and improved surveillance systems will inevitability pick up on more of them as they get more advanced [149,150]. The new variant (B.1.1.529) was identified as a variant of concern and was given the name of Omicron on 26 November 2021 based on the suggestion of the WHO’s Technical Advisory Group on Virus Evolution (TAG-VE) [149,150]. When compared to other variants of the virus, such as Delta, it is not yet known if infection with Omicron causes more severe disease. In South Africa, preliminary data suggests that the rate of hospitalizations is increasing; however, this may be due to an increase in the general number of persons becoming infected rather than a result of a specific infection with Omicron, according to the available data [149]. Besides that, another highly mutated variant that spread rapidly, and was given the IHU name (B.1.640), was reported recently. So far, this variant has been discovered primarily in France, though it has also been discovered in a number of other countries as well [151]. The B.1.640 variant is not a new variant. For at least three months now, it has been detected. The week-old study by researchers from Méditerranée Infection in Marseille, which is part of France’s Instituts hospitalo-universitaires (IHU, or University Hospital Institutes), triggered a sudden discussion about this variant [151]. In comparison to the spread of Omicron, this variant is far less concerning. The most recent detection of this variant was on 25 December 2021, according to the available data. After that, no additional cases have been discovered in the global databases [151]. The exponential versatility of SARS-CoV-2 has hindered the control efforts implanted to reduce its infectivity. The emergence of new variants made current vaccines less effective, even in the recent rapid development and progression of therapeutic approaches, which urges the need to focus more on effective strategies to combat this unprecedented global pandemic.

**Figure 3:**
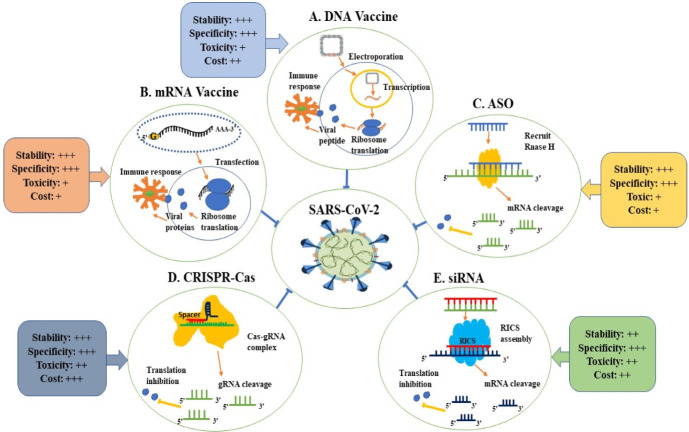
Applications of therapeutic nucleic acids in the fight against SARS-CoV-2. (**A**) DNA vaccine: The SARS-CoV-2 S protein is contained within a circle of DNA, which serves as a DNA vaccine. Following electroporation, the permeation of the cell membrane will be increased, allowing DNA to enter the cytoplasm and so reach the nucleus more easily. Following then, DNA will be transcribed into mRNA, which will be further translated into the SARS-CoV-2 S protein and expressed on the cell membrane. (**B**) mRNA vaccine: The cytoplasmic integration of nanoparticle-encapsulated mRNA expressing the SARS-CoV-2 antigen will take place. The S protein mRNA utilizes the ribosome and bases to translate S proteins, which are expressed on the cell membrane. Immune cells will recognize the membrane S protein, which will result in the activation of an immune response. (**C**) ASO: ASO attaches to the complementary sequence of an mRNA in the nucleus. Following an RNA–DNA hybrid, which becomes a substrate for RNase H, the complementary base pairing between ASO and mRNA results in endonuclease-mediated transcript suppression. The transport of mRNA into the cytoplasm is inhibited, blocking protein synthesis. (**D**) CRISPR–Cas: Upon entering the cell, the Cas protein and the gRNA are both expressed, and the Cas protein and the gRNA form a complex with one another. In this way, the spacer region serves as a guide for the Cas effector by matching to complementary sequences in the viral genome, allowing the associated Cas effector to cleave the viral RNA and disrupt viral functions. (**E**) siRNA: The RISC recognizes and loads the siRNAs, which then separates two strands of associated siRNAs and releases the sense strands. In addition, the antisense strand associated with the RISC directs the complex to the target matching RNA sequences, which could host cellular transcripts or viral RNAs, ultimately leading to RNA degradation mediated by the RISC enzyme. As a result, these siRNAs have the potential to downregulate the expression of the target host/viral genes that are critical for viral activity and to disrupt viral replication and transcription [152,156,157,158,159]. One plus (+) indicates the lowest level, while three pluses (+++) indicate the highest level; these data were compiled from several articles [152,156,157,158,159]. Abbreviation: CRISPR, Clustered Regularly Interspaced Short Palindromic Repeats; Cas, CRISPR-associated; ASO, antisense oligonucleotides; RISC, RNA-induced silencing complexes; siRNA, small interfering RNA; gRNA, guide RNA.

Among the promising strategies to combat SARS-CoV-2 is nucleic acid-based therapeutics. Therapeutic nucleic acids (TNAs) include many pioneering technologies such as antisense oligonucleotides (ASOs), micro RNAs (miRNAs), small interfering RNAs (siRNAs), clustered regularly interspaced short palindromic repeats–CRISPR-associated protein (CRISPR–Cas), and mRNA vaccines [152]. Figure 3 shows the mechanism of action and criteria of each TNAs. TNAs are capable of targeting specific sequences of interest in the viral genome with a focus on the highly conserved sequence, such as targeting the highly conserved RNA-dependent RNA polymerase (*RdRp*) gene in the open reading frame 1ab (ORF1ab) region and the *N* gene by CRISPR-associated RNAs (crRNAs) deployed by the Cas13d system [153]. These antiviral therapeutics can inhibit viral gene expression [154] either during or after transcription [155]. Hence, nucleic acid-based therapeutics present great potential in combating SARS-CoV-2 infection. The most needed feature nowadays is scalable, rapid production; hence, nucleic acid-based therapeutics that possess this feature have the potential to be applied globally. Based on the published articles [152,156,157,158,159], we believe that mRNA vaccines, DNA vaccines, CRISPR, and ASO are more stable than the siRNA approach. Still, there have been no statistically significant comparative studies published to date. Despite that, we believe that the CRISPR systems will be more expensive and possibly harmful than all other approaches in the future. However, we think that ASO and mRNA would be the most cost-effective method.

Based on the endogenous gene silencing mechanism at the translational level, known as RNA interference (RNAi) via small dsRNA molecules [160], which involve RNA-induced silencing complex (RISC) [161], effective therapeutic strategies could be developed against the SARS-CoV-2. However, RNAi application is associated with off-target effects [162] as well as delivery issues [163]. The gene-editing siRNAs are one of the TNAs that can be applied in the fight against SARS-CoV-2. This therapy can knock out the gene of interest without inducing an interferon response [164]. Therefore, a database was developed where it will help develop siRNAs that offer a prediction of off-target binding [165] as well as carriers of nanoparticles or viral vectors were developed, which will help in delivering siRNAs [27]. Nine siRNA, potentially conserved targets were identified bioinformatically in the SARS-COV-2 genome (GenBank accession number, MN908947.3) for safe siRNAs application [166]. Targeting highly conserved regions, the siRNAs Hel1, Hel2, siUC7, and siUTR3 showed potent SARS-CoV-2 gene expression interference delivered via a stable lipid nanoparticle (LNP), known as stealth LNP (sLNP), in the shape of a DOTAP40C or DOTAP/MP3 LNP-siRNAs (dmLNP-siRNAs) formulation [167]. Several siRNAs developed for the SARS-COV virus could potentially be used against SARS-COV-2 (Table 1). However, six designed siRNAs for most of the virus mRNAs (siRNA 1, siRNA 2, siRNA 3, siRNA 4, siRNA 5, and siRNA 6) were reported to have a fair but less than acceptable level of proliferation inhibition [168].

Given that about 2600 human miRNAs have been listed in the miRNA registry (miRBase 22), they are estimated to affect more than 60% of all human protein-coding genes [171,172]. miRNAs are considered a promising TNA in fighting COVID-19 infection, particularly for modifying proteins that are inaccessible to other small molecules [173]. miRNA’s therapeutic mode of action is usually via either blocking cellular receptors, preventing viral replication, or inhibiting the function of the viral proteins [174]. The therapeutic application of miRNA can be in the form of miRNA mimics to interfere with the SARS-CoV-2 gene expression or miRNA inhibitors against SARS-CoV-2-related endogenous miRNAs [173]. The first approach involves using miRNA mimics to reduce the expression of proteins that have been inappropriately induced [173]. Precursor miRNAs (pre-miRNAs) are produced in the nucleus after processing by the dsRNA-specific endonuclease (Drosha) from primary miRNA transcripts. Upon entering the cytoplasm, pre-miRNAs are processed by the Dicer enzyme, resulting in mature miRNA formation [175]. A mature, single-stranded miRNA is coupled with RISC, which binds to the 3′-UTR of the target mRNA and prevents it from being translated [176]. Hence, miRNAs directly affect the translation process by inhibiting the process or causing mRNA degradation. As a result, they can control a wide range of cell processes, including cell differentiation, apoptosis, growth, development, and neurological diseases, by modifying protein levels [177]. In the field of gene silencing, microRNA mimic technology (miR-Mimic) is a promising innovation. The non-natural double-stranded RNA synthesized using this technology is designed to have a 5′ end with a motif that is partially complementary to a specified sequence in the 3′-UTR of the target gene. If this piece of RNA, which mimics an endogenous miRNA, is present in the cell, it can attach specifically to its target gene and cause post-transcriptional inhibition of the target gene [178].

The second approach is based on the development of miRNA antagonists, such as anti-miRs or antagomirs, and inhibitors to increase or rescue the expression of specific proteins that have been downregulated [173]. Antagomirs (also known as blockmirs) are synthetically designed molecules that are used to antagonize miRNAs oligonucleotides. Antagomirs are entirely complementary to the mature miRNA sequence and contain a variety of phosphonothioate moieties to increase their stability [178]. Steric blocking mechanism, as well as hybridization to miRNA, allows these anti-miRs to be used as an additional control and for treatment of certain cellular disorders. If we can identify the miRNA sequences involved in viral infections, the utilization of anti-miRs, as a promising therapy to disrupt the pathways that induce the up- or downregulation of cell proteins that result in the appearance of disease symptoms, will be achieved [178].

Recently, various miRNA-based therapies have shown therapeutic potential in clinical studies [179,180], which is expected to continue in the future. siRNAs are found to be more specific than miRNAs because miRNAs target multiple mRNAs relative to siRNAs, which target only one mRNA [181], but are less efficient than miRNAs [182]. Although miRNAs are susceptible to conserved target sequence mutations [183], the human miRNAs, miR-4699-3p, miR-299-5p, and miR-12132 bind to the altered N protein coding region via 28881-3 GGG/AAC mutations [184]. Hence, miRNA offers a versatile and innovative therapeutic approach, possibly targeting and controlling mutations of concern.

**Figure 4:**
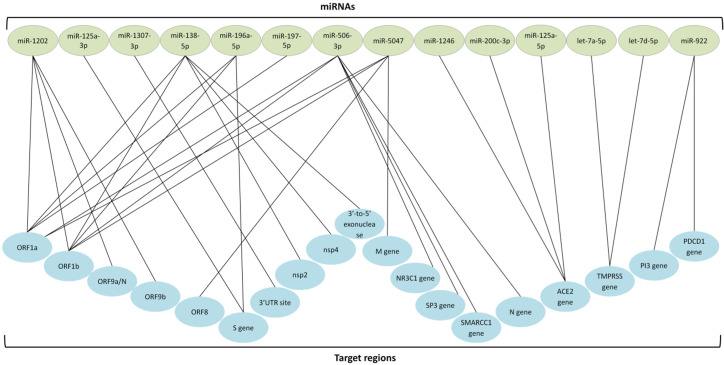
Targeting the SARS-CoV-2 genome via several host miRNAs. Abbreviation: UTR, untranslated region; ORF, open reading frame; S gene, spike gene; nsp, nonstructural protein; M gene, transmembrane gene, NR3C1, glucocorticoid receptor gene; SMARCC1, SWI/SNF related, matrix associated, actin dependent regulator of chromatin subfamily C member 1; N gene, nucleocapsid gene; ACE2, angiotensin-converting enzyme 2; TMPRSS, transmembrane serine protease; PDCD1, programmed cell death protein 1 [153,174,183,185,189,191,193,194,195,196,197,198,199,200,201,202,203,204,205,206].

Several miRNAs, on the other hand, can be used as an effective antiviral agent against SARS-CoV-2. miR-200c inhibited ACE2 receptor expression in cardiomyocytes [185], exhibiting the promise of miRNA therapy. miR-98-5p blocks SARS-CoV-2 entry via TMPRSS2 expression reduction in lung epithelia [186]. miR-32 showed maximum TMPRSS2 gene suppression and significant TMPRSS2 expression reduction by miR-214 and miR-98, offering innovative tools to prevent SARS-CoV-2 entry [187]. Furthermore, 42 conserved miRNAs have been predicted to have antiviral properties against SARS-CoV-2 [188]. One hundred and twenty-eight low-expressed miRNAs in lung tissue are expected to target the SARS-CoV-2 genome and higher expression might suppress the infection [189]. miR-548c-5p, which suppresses the proliferation of colorectal cancer (CRC) [190], has been predicted to bind to 15 sites of the SARS-CoV-2 genome and exert potential therapeutic activity [191]. Multiple deduced antisense miRNAs were hypothesized to disrupt translation via binding to the 3′UTR, 5′UTR, and ORF9 regions of the SARS-CoV-2 genome [192]. Furthermore, the highly expressed miR-16, miR-200, and miR-24 in lung epithelia were highlighted with good prospects of mitigating COVID-19, likely in the form of miRNA mimics, as the former regulates inflammatory mediators and later two miRNAs downregulate ACE2 [173]. Figure 4 summarizes several host miRNAs targeting the SARS-CoV-2 genome or the cellular receptor in the host with COVID-19 therapeutic prospects.

Multiple miRNAs in the host or SARS-CoV-2-encoded are associated with SARS-CoV-2 infection, where some might even inhibit the immune system [207]. These endogenous miRNAs might be a potential target for miRNA inhibitors to suppress SARS-CoV-2 infection. As such, cytokine storms can be reduced or suppressed via targeting the associated miRNAs, such as miR-125b, miR-138, miR-199a, and miR-21 [208]. The exosomal miR-424 expression is significantly elevated in SARS-CoV-2 infection, triggering thrombosis [209], which can also be an ideal target to reduce the concern of thrombotic complications. Numerous host miRNAs were found bioinformatically to interact with the viral genome. Many virus-derived miRNAs interact with the human genome, all of which present the opportunity to be targeted for interfering with SARS-CoV-2 infection [194]. miR-447b could also be a potential target as it binds to S protein RNA and facilitates viral entry [210]. Interestingly, miR-1307-3p was found with the highest expressed level in the lung tissue and showed high affinity to 3′-UTR of the SARS-CoV-2 genome [174], which makes it the model target to suppress the infection. Human miR-122 binds with high affinity to the SARS-CoV-2 genome, presenting a potential target for modulation [211]. Several databases offer insight into the therapeutic potential of miRNAs, such as the therapeutic target database (TTD) for COVID-19 drugs in clinical trials [212], IntaRNA for RNA–RNA interaction prediction [213], miRNA for expression profiles of miRNAs [214], psRNATarget for small RNA analysis [215], microRNA.org for miRNA target prediction [216], and miRTarBase for experimentally validated microRNA–target interactions [217,218]. By targeting host or viral-derived miRNAs and targeting mutated target sequences, the various miRNA strategies provide the optimum platform to be utilized against COVID-19 infection. Hence, miRNA-based therapy has great potential in dealing with the SARS-CoV-2 infection due to its versatile applications.

**Figure 5:**
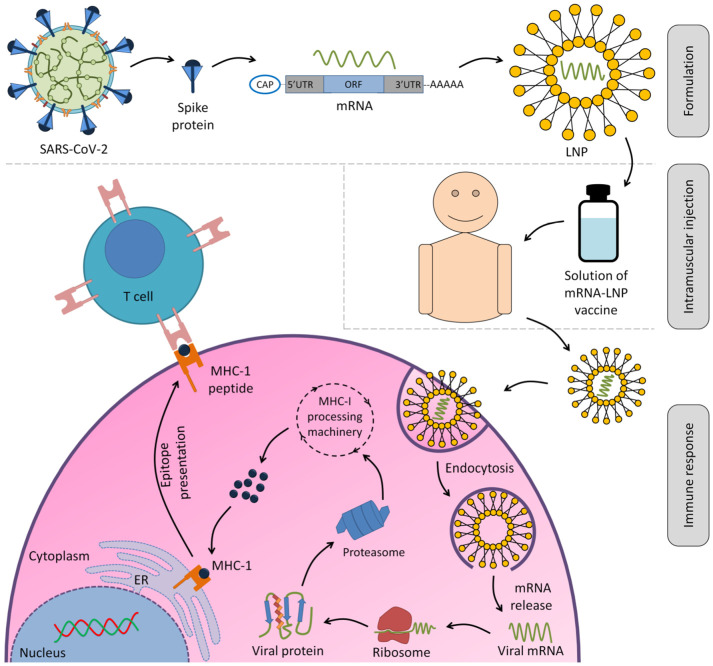
Scheme of the mRNA vaccine’s mechanism of action. The delivery of mRNAs is accomplished via a carrier to improve mRNA distribution and stability in the cell, such as an LNP. Afterwards, the LNP–mRNA vaccines are injected intramuscularly. The mRNAs are released and translated by the host protein synthesis machinery after LNP–mRNAs enter the host cells. The produced proteins are degraded by proteasomes, resulting in peptides that are then linked to MHC I molecules and presented on the surface of host antigen-presenting cells. CD8+ T lymphocytes identify the peptide-MHC I complexes and respond with cellular immunological responses. MHC, major histocompatibility complex; ER, endoplasmic reticulum; UTR, untranslated region; ORF, open reading frame.

mRNA vaccines are considered one of the promising nucleic acid-based therapeutics due to their high potency, rapid, cheap manufacturing, and safe administration features [219]. Their manufacturing does not involve any living part of the organism; it is either conventional via in vitro transcription of plasmid DNA and adding a cap analog and a poly(A) tail or derived from alphavirus RNA replication to form self-amplifying mRNA (SAM) vaccines [220]. mRNA vaccines are synthesized in vitro from a DNA template to express the intended antigen in host cells [221]. The intended potent immune response is developed as mRNA is delivered to the host cell cytoplasm and encodes the desired antigen (Figure 5) [220]. mRNA vaccines have priority over conventional vaccines in many aspects, such as repeated administration [222] and safety concerns of conventional vaccines, especially live-attenuated vaccines, to cause infection [223]. In contrast, mRNA degradation in the cell reduces the potential risk of infection [224]. The enhancement ability of the introduced mRNA structure promotes the antibody lifespan, translation efficacy [225], flexibility, and neutralizing antibodies potency with just two doses [226]. Therefore, mRNA vaccines offer scalable, rapid manufacturing with little platform altering [227], and a fast-responding strategy to overcome the current pandemic dilemma [228]. Unfortunately, these vaccines still have some drawbacks, such as the need to store in extremely low temperatures, which hinders transportation (cold chain challenges) and storage [229], which can be slightly reduced via encapsulation with LNPs [230], escape immune system recognition via viral envelope glycosylation [231], and possible side effects such as local and systemic inflammatory responses [228]. Ultimately, mRNA vaccines still hold one of the keys to stop the SARS-CoV-2 infection.

mRNA-1273 (Moderna COVID-19 vaccine) was one of the first developed mRNA vaccines against SARS-CoV-2, which is currently in Phase III clinical trials, which showed the production of a potent neutralizing antibody response [232] and the vaccine efficacy was reported as 94.1% [82]. This vaccine demonstrated early the effectiveness of mRNA vaccination in the neutralization of SARS-CoV-2. Although the emerging mutations of the S protein in the new variants have shown a small effect against neutralization via two Pfizer–BioNTech (BNT162b2) doses, where elicited, the sera-neutralization geometric mean titers (GMTs) were 0.81- to 1.46-fold against the USA-WA1/2020 virus [233]. A study conducted in Qatar showed an efficacy of 89.5% against the B.1.1.7 variant and 75.0% against the B.1.351 variant [67] as compared to 95% against the Wuhan virus in the USA [42], and 92.6% after the first dose only [234]. However, the BNT162b2 vaccine was shown to be effective against the B.1.1.7 variant [47]. In Israel, a study showed 91.5% efficacy after 14 days after a two-dose vaccination, where the B.1.1.7 variant was prevalent by 94·5% [235]. The BNT162b2 vaccine has been found to have no association with thrombocytopenic, thromboembolic, and hemorrhagic events, unlike the ChAdOx1 vaccine [236]. mRNA vaccines are highly versatile due to the ability to deliver the material of choice and the high degree of manipulation it allows [237]. Hence, mRNA vaccines show great promise by easily adjusting the introduced mRNA to meet the new mutated variants.

Among the other TNA strategies to combat SARS-CoV-2 is the employment of the CRISPR–Cas systems. CRISPR–Cas9 antiviral activity is conducted via expression disruption or direct neutralization of the viral genome [104]. Diagnostic systems have been developed based on the CRISPR system, which offers rapid, precise, and highly sensitive SARS-CoV-2 detection [238]. A prophylactic antiviral CRISPR in human cells (PAC-MAN) strategy was developed to prevent viral replication utilizing Cas13d to neutralize viral RNA [153]. CRISPR–Cas13 is also used in the CARVER (Cas13-aided viral expression and readout restriction) technology to neutralize RNA-based viruses [239]. ASO is another TNA strategy to eliminate the SARS-CoV-2 infection. ASO targets mRNA transcripts, small RNA, or long non-coding RNA [159]. Several ASOs have been designed to target regions of replication and transcription, such as the ORF1a and ORF1b regions in the SARS-CoV-2 genome, to inhibit COVID-19 infection [240]. These strategies can be deployed with other promising approaches, such as miRNA, to neutralize SARS-CoV-2 and reduce the cytokine storm.

## 4. Targeting the Cytokine Storm via Nucleic Acid-Based Approaches

Among the nucleic acid-based approaches, miRNAs hold great potential in attenuating the cytokine storm with versatility and high efficiency. Management of the cytokine storm in the SARS-CoV-2 infection could be accomplished via multiple mechanisms involving miRNAs, such as through miRNAs modulation of the involved inflammatory signaling pathways or even modulating the inflammatory response-related host miRNAs. As such, miRNA mimickers were hypothesized to be feasibly applied as cytokine storm anti-inflammatory agents by targeting the 3′UTR of pro-inflammatory mRNAs [241]. miR-26a-5p, miR-29b-3p, and miR-34a-5p were discovered to play a regulatory role in the endothelial dysfunction and inflammatory response of COVID-19. In addition, miR-26a-5p was reported to downregulate IL-6 and ICAM-1, while miR-29b-3p was found to downregulate IL-4 and IL-8, which are low-expressed during the infection [242]. Therefore, these three miRNAs could be the potential candidates to reduce the cytokine storm, especially miR-26a-5p, which targets IL-6 and present the opportunity to reduce mortality.

Many miRNAs were previously reported to modulate inflammatory mediators. Targeting these miRNAs to regulate the inflammatory response in the cytokine storm or directly applying these miRNAs could give rise to many novel therapeutics to reduce or suppress the cytokine storm and the COVID-19 infection. For instance, the mRNA 3′UTR of IL-1β, IL-6, and IL-8 could be targeted by numerous miRNAs (Table 2) [241]. The most common miRNAs that regulate the COVID-19–ACE2 interaction networks were miR-27a-3p, miR-10b-5p, miR-302c-5p, miR-587, miR-124-3p, and miR-16-5p [243], which offer great potential in reducing the cytokine storm of COVID-19 (Table 2). miR-16-5p was found to target multiple binding sites across coronavirus species [173,244], binds to 15 binding sites on the SARS-CoV-2 genome, is abundant in the alveolar A549 cells [173], regulate the COVID-19–ACE2 interaction networks [243], and downregulate the expression of IL-1β, IL-6, and TNF-α [245,246]; hence, it can be ideal choice to quell the storming cytokines of COVID-19. Another good candidate for the storm might be miR-125a, which downregulates TNF-α [247]. The cytokine storm could be attenuated by targeting the major player in the cytokine induction process, IL-17, via RNAi applications, including miRNA [248]—most notably, miR-129, which targeted human IL-17A, IL-17D, and IL-17RB [249]. MiR-302a reduces the cytokine storm in influenza A virus (IAV) infection [250]; hence, it is considered another good candidate for the COVID-19 cytokine storm attenuation. Even nutraceutical agents were found to possess therapeutic potential in treating the cytokine storm as they can regulate host miRNAs involved in the inflammatory response [251]. In addition, other cytokine downregulatory agents could be considered as potential targets [252], such as miR-146a, which downregulate TNF-α, IL-6, and IL-8; as well as miR-146b, which downregulate IL-1β [253] and miR-199a, which downregulate at least TNF-α, IL-1β, and IL-6 in alveoli [254]. Multiple-host miRNAs induce inflammatory mediators rather than downregulate them, and could be targeted by inhibitors to reduce or eliminate the cytokine storm. MiR-125b was found to elevate the expression of TNF-α and IL-8 [255], which could be a fitting miRNA inhibitor target [252]. One of the promising miRNA inhibitors would be the antagomirs for the safe, scalable, specific, and efficient silencing capability of endogenous miRNAs [252]. Therefore, TNAs, especially miRNA therapeutics, hold the key to quell the cytokine storm and stomping COVID-19.

## 5. Advantages and Disadvantages of NAT

Several COVID-19 vaccines have been authorized to be used by various regulatory bodies across the world since about January 2021. Despite the development of successful vaccinations, a greater research focus on all the therapeutic approaches is necessary, particularly in light of the evolving SARS-CoV-2 variants and potential vaccine-resistant strains [262]. Herein, we demonstrate the advantages and disadvantages of several NAT methods, for their future therapeutic application against SARS-CoV-2 and any new coronavirus variants of concern to global human health, as shown in Table 3.

## 6. Genes Identified as a New Potential Treatment and Protective against COVID-19

According to the findings of an international meta-analysis conducted by researchers at the Karolinska Institutet in Sweden, a particular gene variant that protects against severe COVID-19 infection has been identified [270]. The researchers were able to locate the variant by analyzing people of different ancestries. This achievement demonstrated the importance of conducting clinical trials that involve people of varied ancestries. The G allele of the *2′-5′ oligoadenylate synthetase* (OAS1) gene rs10774671 was reported as having a protective role against COVID-19 hospitalization in those of African ancestry and European ancestry [270,271]. According to the researchers, the length of the protein expressed by the *OAS1* is determined by the protective gene variant (rs10774671-G) [270]. The *OAS* region was discovered to be a COVID-19 risk locus in association studies involving primarily European ancestry individuals [272,273]. The protective haplotype occurring in individuals of European ancestry from Neanderthals is roughly 75 kb in size and spans the three genes, which are *OAS1*, *OAS2*, and *OAS3* [274]. A candidate causative variant in the region is rs10774671, which occurs in the splice acceptor location at exon 7 of *OAS1* and where the protective (G) allele leads to a nearly 60% more active and longer OAS1 enzyme [271,275]. Because this region of DNA contains a large number of genetic variants, it is challenging to identify the specific protective variant that might potentially be used as a target for medical treatment against severe COVID-19 infection in humans [270].

Researchers from the Genetics of Susceptibility and Mortality in Critical Care (GenOMICC) consortium, a global collaboration studying genetics in critical illness, compared the genetic information of patients in intensive care units (ICU) diagnosed with COVID-19 to healthy volunteers’ samples from other current studies, including 100,000 Genomes, Generation Scotland, and UK Biobank [272,276]. The goal of this study was to discover genes that could aid in understanding how COVID-19 causes lung damage at the molecular level. The study done by Pairo-Castineira et al., 2021, analyzed DNA from 2743 ICU participants and discovered changes in 5 genes when compared to the DNA from healthy samples. The genes *DPP9, TYK2, IFNAR2, OAS1*, and *CCR2* may be able to explain why some individuals are associated with severe COVID-19 infection while others have a less severe infection; hence, these genes may be able to predict the effect of possible drug treatments on patients [272,277].

This study found evidence that high expression of *TYK2* or low expression of *IFNAR2* are associated with life-threatening disease using Mendelian randomization, where the low activity of *TYK2* and increased activity of *IFNAR2* can protect against COVID-19 infection [272]. In addition, the high expression of the monocyte–macrophage chemotactic receptor CCR2 was associated with severe COVID-19 using transcriptome-wide association in lung tissue [272]. The findings indicate that, during COVID-19 infection, there are numerous and powerful genetic signals associated with mediators of inflammatory organ damage and major host antiviral defense mechanisms [272]. Targeted treatment with therapies may be possible for both of these mechanisms. Because some genetic variants respond in a similar way to specific drugs, researchers have been able to predict the effect of drug treatments on COVID-19 patients [276,277]. The JAK inhibitors, which include the medicine baricitinib, are a class of anti-inflammatory drugs that are known to decrease the function of the *TYK2* gene; hence, these drugs may have potential in the treatment of COVID-19 patients. In addition, the therapy with interferon proteins released by immune system cells may mimic the effect of increased activity of the gene *INFAR2* in order to protect against COVID-19 virus infection [276,277].

This genetic information provides a roadmap through the complexity of immunological signals, indicating the route to major drug targets, which will quickly indicate which drugs should be at the top of the list for clinical testing, thereby improving the quality of life and prognosis. On the other hand, large-scale randomized clinical trials will be required before any changes in clinical practice can be made. According to the researchers, clinical trials should concentrate on drugs that target these specific anti-inflammatory and anti-viral mechanisms [272].

## 7. Future Directions

To reduce the devastating effects of coronavirus infections and other highly pathogenic virus outbreaks on human life and global healthcare systems, a concerted effort to develop effective drugs and vaccines against current and potential future coronavirus infections, and other highly pathogenic virus outbreaks, is needed. Because clinical drug development is such a costly and time-consuming process, the COVID-19 outbreak emphasizes the necessity of developing broad-spectrum antiviral treatments and using novel techniques, such as artificial intelligence, to speed up drug research. Given the lengthy process of developing new drugs, the current drug repurposing method has emerged as one of the preferred options for treating SARS-CoV-2-infected people. Identification of inhibitors directed at the replication or infection processes associated with SARS-CoV-2 or other similar coronaviruses, as well as the symptomatic consequences of their infections, leading to severe illness and/or death, are long-term therapeutic development goals for the pharmaceutical industry.

More collaboration in the areas of antiviral discovery and clinical trial performance would increase patients’ access to treatment candidates with better therapeutic potential and, perhaps, shorten the time it takes to bring these medicines to market. To address both molecular processes and pharmacological approaches, which will help treat present and future coronavirus epidemics, more intensive effort from research institutes and pharmaceutical businesses is required.

Regardless of the incredible speed of drug development as seen in the wake of the unpredictable SARS-CoV-2 outbreak, this virus still has the priority and proved complicated to control. This unprecedented pandemic requires more effective effort to use every strategy available to find an effective agent against this virus. At present, many prominent therapeutic approaches offer potential antiviral treatment against SARS-CoV-2. Nucleic acid-based approaches or TNAs demonstrate significant potential to eliminate the global dilemma developed by this virus. Among the many TNAs present, miRNAs promise the high efficiency of multiple targets and the versatile application they can offer. siRNAs also could be very promising with their high specificity while delivered using the appropriate nanoparticles. mRNA vaccines offer a scalable, low-cost approach with ease of manipulation of the introduced genetic material. Hence, TNAs present an effective platform to combat the COVID-19 infection as it possesses the required features for swift application of safe administration, rapid, low-cost, scalable production capacity, and high versatility. Mainly, TNAs could be keys to mitigate the cytokine storm and save the lives of the inflicted. Apparently, miRNA therapeutics hold great potential in attenuating the cytokine storm, mainly via mimickers or inhibitors.

## 8. Conclusions

Vaccinating hundreds of millions of people using COVID-19 mRNA vaccines will provide much-needed data on non-viral RNA delivery (especially with the utilization of LPN) and the immune response, safety, and effectiveness of this type of vaccine. Researchers may use these findings to execute an efficient approach by integrating siRNAs, miRNA mimics, antagomirs, or even genes in the same or comparable lipid nanoparticles used in vaccines for therapeutic applications (including regenerative medicine) and gene therapy in the future. Among the TNA-based approaches, miRNAs hold great potential in attenuating the cytokine storm with versatility and high efficiency. Multiple miRNAs in the host or SARS-CoV-2-encoded are associated with COVID-19 infection, where some might even inhibit the immune system. As such, the cytokine storm can be suppressed via targeting the associated miRNAs. Therefore, TNAs, especially miRNA therapeutics, hold the key to quell the cytokine storm and tackling COVID-19. The promising results with the COVID-19 vaccines offer a chance to fulfil the failed initial promise of gene therapy, which aimed to revolutionize molecular medicine while also moving away from viral-based gene delivery. Given the importance of vaccines in the future prevention of coronavirus-related illnesses, several novel techniques are currently in place that is encouraging. It is worth noting that without the earlier preclinical and clinical studies demonstrating the effectiveness of lipid nanoparticles as RNA delivery vehicles, these COVID-19 vaccines would not have been available in time to stop the pandemic.

## Figures and Tables

**Table 1:** Patented siRNAs for the SARS-CoV virus with potential use against SARS-CoV-2.

Patent ID	Description	Publication Number/ Application Number	CAS RN	Target Region	Reference
CN101173275	Two dsRNAs were designed to target two regions of the SARS protein M mRNA	101173275/200610114168.0	1023405-01-7 1023405-02-8	220–241 region of M1 protein	[159]
			1023405-03-9 1023405-04-0	460–480 region of M2 protein	[159]
US20050004063	Six siRNAs were designed to target mRNA sequences of replicase A1 gene region	20050004063/10848737	821121-38-1	512–531 bp of replicase A1	[159,169]
			821121-38-2	586–604 bp of replicase A1	
			821121-38-3	916–934 bp of replicase A1	
			821121-38-4	1194–1213 bp of replicase A1	
			821121-38-5	3028–3046 bp of replicase A1	
			821121-38-6	5024–5042 bp of replicase A1	
CN1569233	siRNAs were designed to target RdRp, helicase, nucleoprotein N, and proteolytic enzymes	1569233/03147398.9	872062-80-1	RdRp	[159,169]
			872062-81-2	Helicase	
			872062-82-3	Nucleoprotein N	
			872067-98-6	Proteolytic enzyme	
CN1648249	siRNAs were specifically designed to target the M, N, and E genes of SARS	1648249/200410016001.1	874840-18-3 874840-32-1	M protein	[169,170]
			874840-19-4 874840-47-8	N protein	
			874840-20-7 874840-46-7	E protein	
CN101113158	Two anti-SARS-CoV siRNA were designed to disturb RdRp gene function of SARS virus	101113158/200610022519.5	-	RdRp	[33,169]
CN101085986	A SARS coronavirus disturbance RNA, which is aimed to disturb RNA for SARS coronavirus and its application, and also to suppress the release of SARS virus by inhibiting the expression of *ORF3a* gene	101085986/200610027475.5	*-*	*ORF3a* gene	[33,169]
WO2017044507	siRNA/nanoparticle formulations for treatment MERS-CoV infection, which was designed to target S protein, RdRp and PL^pro^	WO/2017/044507/PCT/US2016/050590	-	S protein, RdRp and PL^pro^	[33,169]
CN1548054	Two siRNAs or it can be RNAi medicine were used in preventing and treating SARS coronavirus. It is anti-SARS coronavirus transcription and replication polymerases.	1548054/03125172.2	-	Viral mRNA, viral N protein, RNA transcriptase, virion RNA, complementary and RNA polymerase.	[27]
WO2005019410	siRNA molecules and their analogs were designed to target respiratory infections, including SARS coronavirus	WO/2005/019410/PCT/US2004/012730	-	nsp-PP1ab, nsp-PP1a and S protein	[27]
US20070191294	Novel double-stranded siRNA analogs comprising LNA monomers, which induces sequence-specific post-transcriptional gene silencing in many organisms by a process known as RNA interference	20070191294/10550152	-	mRNA, pre-mRNA, or a variety of structural RNAs (such as tRNA, snRNA, scRNA and rRNA) or even regulatory RNAs (like miRNAs).	[27]

PL^pro^, papain-like protease; LNA, locked nucleic acid; MERS-CoV, middle-east respiratory syndrome coronavirus; nsp, nonstructural proteins; PP, polyprotein; RdRp, RNA-dependent RNA polymerase; ORF, open reading frame; siRNA, short interfering; tRNA, transfer RNA; snRNA, small nuclear RNA; scRNA, small conditional RNA; rRNA, ribosomal RNA; miRNAs, microRNAs.

**Table 2:** List of miRNAs that target the 3′UTRs of IL-1β, IL-6, and IL-8 mRNAs, and the recent findings amid the COVID-19 pandemic.

mRNA	miRNA Binding SITES	Sequences of miRNA Binding Sites *	Recent Findings	References
IL-1ß	miR-376c-3p	43-AACAUAGAGGAAAUUCCACGU-63	miR-7 and miR-429 target RPS6KB1 mRNA and inhibit the viral replication.miR-101 and miR-7 target the mTOR mRNA and inhibit the viral replication.miR-21, miR-155 and miR-126 were reported as potential prognostic factor of COVID-19 based on in vivo study miR126-3p and miR-21-5p were reported as potential biomarkers based on in vivo study	[175,178,256,257]
	miR-155-5p	4-UUAAUGCUAAUCGUGAUAGGGGUU-27		
	miR-181c-3p	65-AACCAUCGACCGUUGAGUGGAC-86		
	miR-587	16-UUUCCAUAGGUGAUGAGUCAC-36		
	miR-101-3p	47-UACAGUACUGUGAUAACUGAA-67		
	miR-10b-5p	27-UACCCUGUAGAACCGAAUUUGUG-49		
	miR-126-3p	52-UCGUACCGUGAGUAAUAAUGCG-73		
	miR-128-3p	50-UCACAGUGAACCGGUCUCUUU-70		
	miR-129–2-3p	57-AAGCCCUUACCCCAAAAAGCAU-78		
	miR-203a-3p	65-GUGAAAUGUUUAGGACCACUAG-86		
	miR-34a-5p	22-UGGCAGUGUCUUAGCUGGUUGU-43		
	miR-34c-5p	13-AGGCAGUGUAGUUAGCUGAUUGC-35		
	miR-375-5p	5-GCGACGAGCCCCUCGCACAAACC-27		
	miR-375-3p	40-UUUGUUCGUUCGGCUCGCGUGA-61		
	miR-429	51-UAAUACUGUCUGGUAAAACCGU-72		
	miR-449a	16-UGGCAGUGUAUUGUUAGCUGGU-37		
	miR-7-5p	24-UGGAAGACUAGUGAUUUUGUUGUU-47		
	miR-21-5p	8-UAGCUUAUCAGACUGAUGUUGA-29		
	miR-204-5p	33-UUCCCUUUGUCAUCCUAUGCCU-54		
IL-6	miR-155-5p	4-UUAAUGCUAAUCGUGAUAGGGGUU-27	miR-98-5p targets and inhibits IL-6 gene expression, in turn influencing several proinflammatory cytokines, including TNF-α, IL-1β, and IL-10. miR-7 and miR-16 target RPS6KB1 mRNA and inhibit the viral replication.miR-100, let-7, miR-7 and miR-99a target the mTOR mRNA and inhibit the viral replication. Upregulation of miR-124-3p causes the degradation of Ddx58, thereby leading to a decrease in viral replication. miR-125a-3p inhibits the cleavage of the S gene miR-138-5p inhibits the cleavage of the ORF1a/b polyprotein gene miR-21-3p expressed in respiratory epithelial cells in the trachea and lung tissues, which targets the binding site of 6 different coronavirus, including SARS-CoV-2 and SARS miR-21 and miR-155 were reported as potential prognostic factor of COVID-19 based on in vivo study	[175,178,206,256]
	miR-125a-3p	53-ACAGGUGAGGUUCUUGGGAGCC-74		
	miR-149-5p	15-UCUGGCUCCGUGUCUUCACUCCC-37		
	miR-192-5p	24-CUGACCUAUGAAUUGACAGCC-44		
	miR-590-3p	56-UAAUUUUAUGUAUAAGCUAGU-76		
	miR-100-5p	13-AACCCGUAGAUCCGAACUUGUG-34		
	miR-671-5p	29-AGGAAGCCCUGGAGGGGCUGGAG-51		
	miR-20a-5p	8-UAAAGUGCUUAUAGUGCAGGUAG-30		
	let-7b-5p	6-UGAGGUAGUAGGUUGUGUGGUU-27		
	miR-16-5p	14-UAGCAGCACGUAAAUAUUGGCG-35		
	miR-376a-5p	7-GUAGAUUCUCCUUCUAUGAGUA-28		
	miR-335-5p	16-UCAAGAGCAAUAACGAAAAAUGU-38		
	miR-98-5p	22-UGAGGUAGUAAGUUGUAUUGUU-43		
	miR-124-3p	53-UAAGGCACGCGGUGAAUGCCAA-74		
	miR-1-3p	53-UGGAAUGUAAAGAAGUAUGUAU-74		
	miR-34a-5p	22-UGGCAGUGUCUUAGCUGGUUGU-43		
	miR-99a-5p	13-AACCCGUAGAUCCGAUCUUGUG-34		
	miR-191-5p	16-CAACGGAAUCCCAAAAGCAGCUG-38		
	miR-128-3p	50-UCACAGUGAACCGGUCUCUUU-70		
	miR-138-5p	10-AGCUGGUGUUGUGAAUCAGGCCG-32		
	miR-182-5p	23-UUUGGCAAUGGUAGAACUCACACU-46		
	miR-195-5p	15-UAGCAGCACAGAAAUAUUGGC-35		
	miR-203a-3p	65-GUGAAAUGUUUAGGACCACUAG-86		
	miR-205-5p	34-UCCUUCAUUCCACCGGAGUCUG-55		
	miR-21-3p	46-CAACACCAGUCGAUGGGCUGU-66		
	miR-21-5p	8-UAGCUUAUCAGACUGAUGUUGA-29		
	miR-221-3p	65-AGCUACAUUGUCUGCUGGGUUUC-87		
	miR-27a-3p	51-UUCACAGUGGCUAAGUUCCGC-71		
	miR-27a-5p	10-AGGGCUUAGCUGCUUGUGAGCA-31		
	miR-330-3p	57-GCAAAGCACACGGCCUGCAGAGA-79		
	miR-34b-5p	13-UAGGCAGUGUCAUUAGCUGAUUG-35		
	miR-375-5p	5-GCGACGAGCCCCUCGCACAAACC-27		
	miR-375-3p	40-UUUGUUCGUUCGGCUCGCGUGA-61		
	miR-429	51-UAAUACUGUCUGGUAAAACCGU-72		
	miR-7-5p	24-UGGAAGACUAGUGAUUUUGUUGUU-47		
	miR-373-3p	44-GAAGUGCUUCGAUUUUGGGGUGU-66		
	miR-372-3p	42-AAAGUGCUGCGACAUUUGAGCGU-64		
	miR-302a-3p	44-UAAGUGCUUCCAUGUUUUGGUGA-66		
	miR-148b-3p	63-UCAGUGCAUCACAGAACUUUGU-84		
	miR-133a-3p	53-UUUGGUCCCCUUCAACCAGCUG-74		
	miR-122-5p	15-UGGAGUGUGACAAUGGUGUUUG-36		
IL-8	miR-195-5p	15-UAGCAGCACAGAAAUAUUGGC-35	Upregulation of miR-17 and miR-214 have an antiviral effect by binding to S-protein-encoding mRNA, hence cause inhibition of the viral replication. miRNA-145 downregulates ADAM17, which is a target of Jagged1/Notch1 signaling in vascular smooth muscle cells. miR-7, miR-17, miR-16 and miR-107 target RPS6KB1 mRNA and inhibit the viral replication. miR-101, let-7, miR-107, miR-7 and miR-99b target the mTOR mRNA and inhibit the viral replication. Upregulation of miR-124-3p causes the degradation of Ddx58, thereby leading to a decrease in viral replication. miR-99b-5p regulates immune reactions. miR-130a predicts the targets of QFPD, and QFPD, which may exert anti-SARS-CoV-2 activity miR-17-5p have antiviral roles against SARS-CoV-2 by targeting the ORF1ab and the S region miR-23a-3p has prognostic and therapeutic effects based on in vivo study miR-146a-5p was reported as potential biomarker based on in vivo study miR-29a-3p promote IL-8 and other pro-inflammatory cytokine expression, despite being inversely correlated with IL-8 in COVID-19	[175,178,193,242,257,258,259,260,261]
	miR-20a-5p	8-UAAAGUGCUUAUAGUGCAGGUAG-30		
	miR-106a-5p	13-AAAAGUGCUUACAGUGCAGGUAG-35		
	miR-17-5p	14-CAAAGUGCUUACAGUGCAGGUAG-36		
	miR-30c-1-3p	56-CUGGGAGAGGGUUGUUUACUCC-77		
	miR-93-5p	11-CAAAGUGCUGUUCGUGCAGGUAG-33		
	miR-373-3p	44-GAAGUGCUUCGAUUUUGGGGUGU-66		
	miR-520c-3p	54-AAAGUGCUUCCUUUUAGAGGGU-75		
	miR-10a-3p	63-CAAAUUCGUAUCUAGGGGAAUA-84		
	miR-1225-5p	1-GUGGGUACGGCCCAGUGGGGGG-22		
	miR-23a-3p	45-AUCACAUUGCCAGGGAUUUCC-65		
	miR-23b-3p	58-AUCACAUUGCCAGGGAUUACCAC-80		
	miR-296-3p	48-GAGGGUUGGGUGGAGGCUCUCC-69		
	miR-302c-5p	8-UUUAACAUGGGGGUACCUGCUG-29		
	miR-302d-5p	6-ACUUUAACAUGGAGGCACUUGC-27		
	miR-450a-5p	18-UUUUGCGAUGUGUUCCUAAUAU-39		
	miR-493-5p	16-UUGUACAUGGUAGGCUUUCAUU-37		
	miR-499a-3p	70-AACAUCACAGCAAGUCUGUGCU-91		
	miR-519d-3p	54-CAAAGUGCCUCCCUUUAGAGUG-75		
	miR-520a-3p	53-AAAGUGCUUCCCUUUGGACUGU-74		
	miR-526b-3p	51-GAAAGUGCUUCCUUUUAGAGGC-72		
	miR-5582-3p	47-UAAAACUUUAAGUGUGCCUAGG-68		
	miR-587	16-UUUCCAUAGGUGAUGAGUCAC-36		
	miR-664a-3p	49-UAUUCAUUUAUCCCCAGCCUACA-71		
	miR-1-3p	53-UGGAAUGUAAAGAAGUAUGUAU-74		
	miR-429	51-UAAUACUGUCUGGUAAAACCGU-72		
	miR-34a-5p	22-UGGCAGUGUCUUAGCUGGUUGU-43		
	miR-155-5p	4-UUAAUGCUAAUCGUGAUAGGGGUU-27		
	let-7b-5p	6-UGAGGUAGUAGGUUGUGUGGUU-27		
	miR-124-3p	53-UAAGGCACGCGGUGAAUGCCAA-74		
	miR-126-3p	52-UCGUACCGUGAGUAAUAAUGCG-73		
	miR-16-5p	14-UAGCAGCACGUAAAUAUUGGCG-35		
	miR-27a-3p	51-UUCACAGUGGCUAAGUUCCGC-71		
	miR-335-5p	16-UCAAGAGCAAUAACGAAAAAUGU-38		
	miR-1291	14-UGGCCCUGACUGAAGACCAGCAGU-37		
	miR-138-5p	10-AGCUGGUGUUGUGAAUCAGGCCG-32		
	miR-101-3p	47-UACAGUACUGUGAUAACUGAA-67		
	miR-107	50-AGCAGCAUUGUACAGGGCUAUCA-72		
	miR-129–2-3p	57-AAGCCCUUACCCCAAAAAGCAU-78		
	miR-130a-3p	55-CAGUGCAAUGUUAAAAGGGCAU-76		
	miR-146a-5p	21-UGAGAACUGAAUUCCAUGGGUU-42		
	miR-147a	47-GUGUGUGGAAAUGCUUCUGC-66		
	miR-194-5p	15-UGUAACAGCAACUCCAUGUGGA-36		
	miR-203a-3p	65-GUGAAAUGUUUAGGACCACUAG-86		
	miR-21-3p	46-CAACACCAGUCGAUGGGCUGU-66		
	miR-21-5p	8-UAGCUUAUCAGACUGAUGUUGA-29		
	miR-210-3p	66-CUGUGCGUGUGACAGCGGCUGA-87		
	miR-212-3p	71-UAACAGUCUCCAGUCACGGCC-91		
	miR-214-3p	71-ACAGCAGGCACAGACAGGCAGU-92		
	miR-221-3p	65-AGCUACAUUGUCUGCUGGGUUUC-87		
	miR-29a-5p	4-ACUGAUUUCUUUUGGUGUUCAG-25		
	miR-29a-3p	42-UAGCACCAUCUGAAAUCGGUUA-63		
	miR-30d-5p	6-UGUAAACAUCCCCGACUGGAAG-27		
	miR-376a-5p	7-GUAGAUUCUCCUUCUAUGAGUA-28		
	miR-671-5p	29-AGGAAGCCCUGGAGGGGCUGGAG-51		
	miR-7-5p	24-UGGAAGACUAGUGAUUUUGUUGUU-47		
	miR-941	47-CACCCGGCUGUGUGCACAUGUGC-69		
	miR-99b-5p	7-CACCCGUAGAACCGACCUUGCG-28		
	miR-520f-3p	55-AAGUGCUUCCUUUUAGAGGGUU-76		
	miR-372-3p	42-AAAGUGCUGCGACAUUUGAGCGU-64		
	miR-148b-3p	63-UCAGUGCAUCACAGAACUUUGU-84		
	miR-133a-3p	53-UUUGGUCCCCUUCAACCAGCUG-74		
	miR-9-5p	16-UCUUUGGUUAUCUAGCUGUAUGA-38		
	miR-30a-5p	6-UGUAAACAUCCUCGACUGGAAG-27		

* All sequences of the miRNA binding site were retrieved from https://mirbase.org/ (Accessed on 19 January 2022). Abbreviations: RPS6KB1, ribosomal protein S6 kinase B1; mTOR, mammalian target of rapamycin; ORF, open reading frame; ADAM 17, a disintegrin and metalloprotease 17; QFPD, Qingfei Paidu Decoction.

**Table 3:** Advantages and disadvantages of several NATs.

Nucleic Acid Therapy	Advantages	Disadvantages	References
mRNA vaccine	Noninfectious, no genome integration risk, reusable platform, simple formulations, rapid and scalable production. It is flexible and can mimic the antigen structure and expression as seen in the course of a natural infection. The anti-vector immunity is avoided. The immunogenicity of the mRNA can be minimized. Alterations can be made to increase the stability of these vaccines. The translation of mRNA occurs in the host cell’s cytosol, averting the risk of any sort of integration into the host genome. Small size, simpler constructs, and lack any extra encoded proteins. The potential risk of infection and insertion induced mutagenesis are minimized by mRNA-based vaccines. Stimulate innate immune response, induction of T and B cell immune response. The self-amplifying mRNA-based vaccines (SAM) vaccine technology is capable of swift and cost-effective vaccines production. These can be used for both therapeutic and prophylactic purposes, as shown by various preclinical and clinical projects.	It needs an efficient delivery and expression into the cytoplasm, due to the plasma membrane hinders the entry of mRNA. Safety issues with reactogenicity have been reported for various RNA based vaccines. Local and systemic inflammatory responses, possible development of autoreactive antibodies, persistence, and bio-distribution of induced immunogenic responses. Toxic effect of delivery system components and non-native nucleotide May need extremely low temperatures for storage and transportation. Mutation in the S protein increases the possibility that the vaccine will not be very effective in the long term. The price of mRNA vaccines is higher (US$30−40 per shot) than conventional or viral vector-based vaccines (US$2−5).	[13,33,112,116,263,264]
DNA vaccine	Fast to produce, scalable, noninfectious and reusable platform. DNA vaccine encodes the antigen and an adjuvant, will induces the adaptive immune response. Stimulating effective humoral as well as cell-mediated immune responses. The synthetic DNA is temperature stable and cold-chain free. It can be developed at an accelerated pace. It does not require the handling of the infectious viral particle. Stable at room temperature. DNA vaccines have low production cost when compared to protein vaccines. Enhanced stability for transportation and storage and can be administered to immunocompromised patients.	It may need special delivery devices. Though it elicits both Cytotoxic and humoral immunity, the titer remains low. Insertion of foreign DNA into the host genome may cause abnormalities in the cell. May induce the antibody production against itself. In human and large animals, DNA vaccine causes lower immunogenicity in comparison to inactivated vaccines. Autoimmune responses and DNA integration in the host genome may occur. Require multiple booster doses.	[13,33,112,263,265]
ASO-based therapy	Acquisition of the target sequence provides immediate knowledge of putative complementary oligonucleotide therapeutics. They can be used for antigen modification of whole-cell immunogens or as a vaccine adjuvant by enhancing the host immune response. Block the expression of specific target genes via complementary hybridization to mRNA. Have high specificity and a well-known mechanism of action.	Insufficient stability and low cellular delivery have not been sufficiently resolved to achieve effective and safe ASO-based vaccines. ASOs bioavailability and the occurrence of potential off-target effects. The possibility of toxicity by immune overstimulation needs to be deeply studied.	[240,266,267]
miRNAs-based therapy	Promising approach against heart failure or cardiovascular diseases, neurological disorders, tumorigenesis, and viral infection. Promising therapeutic avenue for future antiviral applications, where it will facilitate the diagnosis and improve the treatment of COVID-19 infection. Potent epigenetic regulators which stimulate the innate and adaptive immune system. miRNAs-based therapeutics could be used in the nanovaccines that are specific with minimal off-target effects.	Less specific due to miRNAs- mediated therapeutics as miRNAs can hybridize with mRNA having partially complementary sequences Can target an entire immune pathway. Various miRNAs with multi-step maturation need several strategies to enhance miRNA’s function and develop miRNAs-based therapeutics against COVID-19. May cause adverse effects on the host genome. May target several signaling pathways and critical cellular processes. Unstable nature of RNA molecules The requirement for targeted delivery based on its site of action.	[33,173,208,268]
siRNAs-based therapy	It does not require translation of mRNA, is programmable, scalable, stable, and able to repress coronaviruses potently. siRNAs are more specific than miRNAs- mediated therapeutics as miRNAs have then the ability to hybridize with mRNA having partially complementary sequences. It has also been shown to be an effective adjuvant for DNA vaccines. Therapeutically effective ASOs are heavily modified, so they do not require a delivery carrier. This limited downstream processing during manufacturing decreases production-associated costs. Allows for addressing the root cause of the infection rather than palliating only the symptoms of the disease both in prophylactic or curative settings Low cost of production to manufacture and easy to design	A siRNA against the Leader sequence was relatively less effective (∼50%, estimated). Limited effectiveness and potential toxicity effects in the early applications. Their efficient delivery to relevant cells in the lung is the next hurdle.	[33,165,167,240,248,264,265,269]
CRISPR–Cas based therapy	Strong antiviral and diagnostic technology platform for a wide variety of ssRNA viruses. Can be used as therapeutic tools for SARS-CoV-2 infection. Can target different regions of a virus or different coronavirus strains simultaneously with the crRNA pool, preventing possible viral escapes. Promising strategy to combat pan-coronaviruses that will occur in pandemics. Can successfully target and cleave the RNA sequences of SARS-CoV-2 fragments and IAV in lung epithelial cell cultures. It is a genetic strategy that can target conserved regions, hence prevent the virus to escape inhibition through mutation. RNA-targeting CRISPR effectors could offer a future promising possible antiviral approach. Cas13 could be programmed to target and destroy a wide variety of mammalian viruses.	The presence of pre-existing antibodies to CRISPRs33. The need to translate the packaged CRISPR mRNA and gRNA in virus infected cells will hinder the clinical translation of this approach. CRISPR systems may be more costly and perhaps toxic than all other systems. Further validation in animal models earlier than clinical checks in humans is required.	[153,159,167,239]

## Supplementary Materials

The following are available online at https://www.mdpi.com/article/10.3390/jpm12030386/s1, Table S1: List of RNA-based COVID-19 vaccine candidates; Table S2: List of RNA-based treatments against COVID-19; Table S3: DNA-based vaccine candidates against COVID-19.

## References

[1] Al-Bsheish M., Jarrar M.T., Scarbrough A. (2021). A Public Safety Compliance Model of Safety Behaviors in the Age of the COVID-19 Pandemic. INQUIRY J. Health Care Organ. Provis. Financ..

[2] Al-Hatamleh M.A.I., Hatmal M.M., Sattar K., Ahmad S., Mustafa M.Z., Bittencourt M.C., Mohamud R. (2020). Antiviral and Immunomodulatory Effects of Phytochemicals from Honey against COVID-19: Potential Mechanisms of Action and Future Directions. Molecules.

[3] Ahmed N., Rizvi A., Naeem A., Saleem W., Ahmed A., Parveen S., Ilyas M. (2020). COVID-19 and public awareness. Prof. Med. J..

[4] Sahul Hameed A.S., Ninawe A.S., Nakai T., Chi S.C., Johnson K.L. (2019). ICTV virus taxonomy profile: *Nodaviridae*. J. Gen. Virol..

[5] Xu R.H., He J.F., Evans M.R., Peng G.W., Field H.E., Yu D.W., Lee C.K., Luo H.M., Lin W.S., Lin P. (2004). Epidemiologic clues to SARS origin in China. Emerg. Infect. Dis..

[6] Zumla A.I., Memish Z.A. (2014). Middle East respiratory syndrome coronavirus: Epidemic potential or a storm in a teacup?. Eur. Respir. J..

[7] Ali Z., Jatoi M.A., Al-Wraikat M., Ahmed N., Li J. (2021). Time to Enhance Immunity via Functional Foods and Supplements: Hope for SARS-CoV-2 Outbreak. Altern. Ther. Health Med..

[8] Zhu N., Zhang D., Wang W., Li X., Yang B., Song J., Zhao X., Huang B., Shi W., Lu R. (2020). A novel coronavirus from patients with pneumonia in China, 2019. N. Engl. J. Med..

[9] Hatmal M.M., Alshaer W., Al-Hatamleh M.A.I., Hatmal M., Smadi O., Taha M.O., Oweida A.J., Boer J.C., Mohamud R., Plebanski M. (2020). Comprehensive Structural and Molecular Comparison of Spike Proteins of SARS-CoV-2, SARS-CoV and MERS-CoV, and Their Interactions with ACE2. Cells.

[10] Fehr A.R., Perlman S. (2015). Coronaviruses: An Overview of Their Replication and Pathogenesis.

[11] Chen R.E., Zhang X., Case J.B., Winkler E.S., Liu Y., VanBlargan L.A., Liu J., Errico J.M., Xie X., Suryadevara N. (2021). Resistance of SARS-CoV-2 variants to neutralization by monoclonal and serum-derived polyclonal antibodies. Nat. Med..

[12] Kubo H., Yamada Y.K., Taguchi F. (1994). Localization of neutralizing epitopes and the receptor-binding site within the amino-terminal 330 amino acids of the murine coronavirus spike protein. J. Virol..

[13] Li Y., Tenchov R., Smoot J., Liu C., Watkins S., Zhou Q. (2021). A Comprehensive Review of the Global Efforts on COVID-19 Vaccine Development. ACS Cent. Sci..

[14] Anis S., Khan M.M., Ali Z., Khan A., Arsalan H.M., Naeem S., Saleem I., Qamar S., Khan M.M., Ahmad A. (2021). Novel corona virus disease (COVID-19): An updated review on epidemiology, pathogenicity, clinical course, treatments, migrant health concerns and risk factors predictions. Pak. J. Pharm. Sci.

[15] Simko F., Baka T. (2021). Angiotensin-converting enzyme inhibitors and angiotensin II receptor blockers: Potential allies in the COVID-19 pandemic instead of a threat?. Clin. Sci..

[16] Bosch B.J., van der Zee R., de Haan C.A.M., Rottier P.J.M. (2003). The coronavirus spike protein is a class I virus fusion protein: Structural and functional characterization of the fusion core complex. J. Virol..

[17] Gunst J.D., Staerke N.B., Pahus M.H., Kristensen L.H., Bodilsen J., Lohse N., Dalgaard L.S., Brønnum D., Fröbert O., Hønge B. (2021). Efficacy of the TMPRSS2 inhibitor camostat mesilate in patients hospitalized with COVID-19-a double-blind randomized controlled trial. EClinicalMedicine.

[18] Hoffmann M., Kleine-Weber H., Schroeder S., Krüger N., Herrler T., Erichsen S., Schiergens T.S., Herrler G., Wu N.H., Nitsche A. (2020). SARS-CoV-2 cell entry depends on ACE2 and TMPRSS2 and is blocked by a clinically proven protease inhibitor. Cell.

[19] Sawicki S.G., Sawicki D.L., Siddell S.G. (2007). A contemporary view of coronavirus transcription. J. Virol..

[20] Krijnse-Locker J., Ericsson M., Rottier P.J.M., Griffiths G. (1994). Characterization of the budding compartment of mouse hepatitis virus: Evidence that transport from the RER to the Golgi complex requires only one vesicular transport step. J. Cell Biol..

[21] de Haan C.A.M., Rottier P.J.M. (2005). Molecular interactions in the assembly of coronaviruses. Adv. Virus Res..

[22] AbuSalah M.A.H., Gan S.H., Al-Hatamleh M.A.I., Irekeola A.A., Shueb R.H., Yean Yean C. (2020). Recent Advances in Diagnostic Approaches for Epstein-Barr Virus. Pathogens.

[23] Wang W., Xu Y., Gao R., Lu R., Han K., Wu G., Tan W. (2020). Detection of SARS-CoV-2 in different types of clinical specimens. JAMA.

[24] Salah M.A.H.A., Binti Hassan S.A., Mat Lazim N., Abdullah B., Binti Wan Sohaimi W.F., Husin A., Cheng K.Y., Yean C.Y. (2021). Design of InnoPrimers-Duplex Real-Time PCR for Detection and Treatment Response Prediction of EBV-Associated Nasopharyngeal Carcinoma Circulating Genetic Biomarker. Diagnostics.

[25] Rahimi A., Mirzazadeh A., Tavakolpour S. (2021). Genetics and genomics of SARS-CoV-2: A review of the literature with the special focus on genetic diversity and SARS-CoV-2 genome detection. Genomics.

[26] Al-Hatamleh M.A.I., Hatmal M.M., Alshaer W., Rahman E., Mohd-Zahid M.H., Alhaj-Qasem D.M., Yean C.Y., Alias I.Z., Jaafar J., Ferji K. (2021). COVID-19 infection and nanomedicine applications for development of vaccines and therapeutics: An overview and future perspectives based on polymersomes. Eur. J. Pharmacol..

[27] Ghosh S., Firdous S.M., Nath A. (2020). siRNA could be a potential therapy for COVID-19. EXCLI J..

[28] Beigel J.H., Tomashek K.M., Dodd L.E., Mehta A.K., Zingman B.S., Kalil A.C., Hohmann E., Chu H.Y., Luetkemeyer A., Kline S. (2020). Remdesivir for the treatment of COVID-19—Final report. N. Engl. J. Med..

[29] Keshavarzi Arshadi A., Webb J., Salem M., Cruz E., Calad-Thomson S., Ghadirian N., Collins J., Diez-Cecilia E., Kelly B., Goodarzi H. (2020). Artificial Intelligence for COVID-19 Drug Discovery and Vaccine Development. Front. Artif. Intell..

[30] Milken Institute (2020). COVID-19 Treatment and Vaccine Tracker. https://milkeninstitute.org/sites/default/files/2020-03/Covid19%20Tracker_WEB.pdf.

[31] Silveira M.M., Moreira G., Mendonca M. (2021). DNA vaccines against COVID-19: Perspectives and challenges. Life Sci..

[32] Liu M.A. (2019). A Comparison of Plasmid DNA and mRNA as Vaccine Technologies. Vaccines.

[33] Piyush R., Rajarshi K., Chatterjee A., Khan R., Ray S. (2020). Nucleic acid-based therapy for coronavirus disease 2019. Heliyon.

[34] Geall A.J., Mandl C.W., Ulmer J.B. (2013). RNA: The new revolution in nucleic acid vaccines. Semin. Immunol..

[35] Restifo N.P., Ying H., Hwang L., Leitner W.W. (2000). The promise of nucleic acid vaccines. Gene Ther..

[36] Rose A., Triano C., Alatovic J., Maas S. Pfizer and BioNTech Announce Early Positive Data from an Ongoing Phase 1/2 Study of mRNA-Based Vaccine Candidate against SARS-CoV-2. https://www.pfizer.com/news/press-release/press-release-detail/pfizer-and-biontech-announce-early-positive-data-ongoing-0.

[37] Mulligan M.J., Lyke K.E., Kitchin N., Absalon J., Gurtman A., Lockhart S., Neuzil K., Raabe V., Bailey R., Swanson K.A. (2020). Phase I/II study of COVID-19 RNA vaccine BNT162b1 in adults. Nature.

[38] Sahin U., Muik A., Derhovanessian E., Vogler I., Kranz L.M., Vormehr M., Baum A., Pascal K., Quandt J., Maurus D. (2020). Concurrent human antibody and TH1 type T-cell responses elicited by a COVID-19 RNA vaccine. medRxiv.

[39] Walsh E.E., Frenck R., Falsey A.R., Kitchin N., Absalon J., Gurtman A., Lockhart S., Neuzil K., Mulligan M.J., Bailey R. (2020). RNA-based COVID-19 vaccine BNT162b2 selected for a pivotal efficacy study. medRxiv.

[40] Sahin U., Muik A., Derhovanessian E., Vogler I., Kranz L.M., Vormehr M., Baum A., Pascal K., Quandt J., Maurus D. (2020). COVID-19 vaccine BNT162b1 elicits human antibody and TH 1 T cell responses. Nature.

[41] Walsh E.E., Frenck R.W., Falsey A.R., Kitchin N., Absalon J., Gurtman A., Lockhart S., Neuzil K., Mulligan M.J., Bailey R. (2020). Safety and immunogenicity of two RNA-based COVID-19 vaccine candidates. N. Engl. J. Med..

[42] Polack F.P., Thomas S.J., Kitchin N., Absalon J., Gurtman A., Lockhart S., Perez J.L., Marc G.P., Moreira E.D., Zerbini C. (2020). Safety and efficacy of the BNT162b2 mRNA COVID-19 vaccine. N. Engl. J. Med..

[43] Vogel A.B., Kanevsky I., Che Y., Swanson K.A., Muik A., Vormehr M., Kranz L.M., Walzer K.C., Hein S., Güler A. (2020). BNT162b vaccines are immunogenic and protect non-human primates against SARS-CoV-2. bioRxiv.

[44] Sahin U., Muik A., Vogler I., Derhovanessian E., Kranz L.M., Vormehr M., Quandt J., Bidmon N., Ulges A., Baum A. (2020). BNT162b2 induces SARS-CoV-2-neutralising antibodies and T cells in humans. medRxiv.

[45] Widger A., Triano C., Alatovic J., Maas S. Pfizer and BioNTech Provide Data from German Phase 1/2 Study Further Characterizing Immune Response Following Immunization with Lead COVID-19 Vaccine Candidate BNT162b2. https://www.pfizer.com/news/press-release/press-release-detail/pfizer-and-biontech-provide-data-german-phase-12-study.

[46] Xie X., Zou J., Fontes-Garfias C.R., Xia H., Swanson K.A., Cutler M., Cooper D., Menachery V.D., Weaver S., Dormitzer P.R. (2021). Neutralization of N501Y mutant SARS-CoV-2 by BNT162b2 vaccine-elicited sera. bioRxiv.

[47] Muik A., Wallisch A.-K., Sänger B., Swanson K.A., Mühl J., Chen W., Cai H., Maurus D., Sarkar R., Türeci Ö. (2021). Neutralization of SARS-CoV-2 lineage B. 1.1. 7 pseudovirus by BNT162b2 vaccine–elicited human sera. Science.

[48] Zhu F., Li J., Hui A., Zhang X., Yang Y., Tang R., Ye H., Ji R., Lin M., Zhu Z. (2021). First report demonstrating the safety and immunogenicity of the SARS-COV-2 BNT162b1 mRNA vaccine in younger and older Chinese adults: A randomized, placebo-controlled, observer-blind Phase I study. Nat. Med..

[49] Amy R., Triano C., Alatovic J., Maas S. Real-World Evidence Confirms High Effectiveness of Pfizer-BioNTech COVID-19 Vaccine and Profound Public Health Impact of Vaccination One Year after Pandemic Declared. https://investors.biontech.de/news-releases/news-release-details/real-world-evidence-confirms-high-effectiveness-pfizer-biontech.

[50] Pitts J., Triano C., Alatovic J., Maas S. Pfizer-BioNTech Announce Positive Topline Results of Pivotal COVID-19 Vaccine Study in Adolescents. https://www.pfizer.com/news/press-release/press-release-detail/pfizer-biontech-announce-positive-topline-results-pivotal.

[51] Pitts J., Triano C., Alatovic J., Maas S. Pfizer and BioNTech Confirm High Efficacy and No Serious Safety Concerns Through Up to Six Months Following Second Dose in Updated Topline Analysis of Landmark COVID-19 Vaccine Study. https://www.businesswire.com/news/home/20210401005365/en/Pfizer-and-BioNTech-Confirm-High-Efficacy-and-No-Serious-Safety-Concerns-Through-Up-to-Six-Months-Following-Second-Dose-in-Updated-Topline-Analysis-of-Landmark-COVID-19-Vaccine-Study.

[52] Hussey C., Budwick D., Talukdar L. Moderna Announces Positive Interim Phase 1 Data for its mRNA Vaccine (mRNA-1273) Against Novel Coronavirus. https://investors.modernatx.com/news-releases/news-release-details/moderna-announces-positive-interim-phase-1-data-its-mrna-vaccine.

[53] Jackson L.A., Anderson E.J., Rouphael N.G., Roberts P.C., Makhene M., Coler R.N., McCullough M.P., Chappell J.D., Denison M.R., Stevens L.J. (2020). An mRNA vaccine against SARS-CoV-2—Preliminary report. N. Engl. J. Med..

[54] Anderson E.J., Rouphael N.G., Widge A.T., Jackson L.A., Roberts P.C., Makhene M., Chappell J.D., Denison M.R., Stevens L.J., Pruijssers A.J. (2020). Safety and immunogenicity of SARS-CoV-2 mRNA-1273 vaccine in older adults. N. Engl. J. Med..

[55] Hussey C., Talukdar L. Moderna’s COVID-19 Vaccine Candidate Meets its Primary Efficacy Endpoint in the First Interim Analysis of the Phase 3 COVE Study. https://www.businesswire.com/news/home/20201116005608/en/Moderna%E2%80%99s-COVID-19-Vaccine-Candidate-Meets-its-Primary-Efficacy-Endpoint-in-the-First-Interim-Analysis-of-the-Phase-3-COVE-Study.

[56] Widge A.T., Rouphael N.G., Jackson L.A., Anderson E.J., Roberts P.C., Makhene M., Chappell J.D., Denison M.R., Stevens L.J., Pruijssers A.J. (2021). Durability of responses after SARS-CoV-2 mRNA-1273 vaccination. N. Engl. J. Med..

[57] Wu K., Werner A.P., Moliva J.I., Koch M., Choi A., Stewart-Jones G.B., Bennett H., Boyoglu-Barnum S., Shi W., Graham B.S. (2021). mRNA-1273 vaccine induces neutralizing antibodies against spike mutants from global SARS-CoV-2 variants. bioRxiv.

[58] Doria-Rose N., Suthar M.S., Makowski M., O’Connell S., McDermott A.B., Flach B., Ledgerwood J.E., Mascola J.R., Graham B.S., Lin B.C. (2021). Antibody persistence through 6 months after the second dose of mRNA-1273 vaccine for COVID-19. N. Engl. J. Med..

[59] Wu K., Choi A., Koch M., Ma L., Hill A., Nunna N., Huang W., Oestreicher J., Colpitts T., Bennett H. (2021). Preliminary analysis of safety and immunogenicity of a SARS-CoV-2 variant vaccine booster. medRxiv.

[60] Choi A., Koch M., Wu K., Dixon G., Oestreicher J., Legault H., Stewart G.B., Colpitts T., Pajon R., Bennett H. (2021). Serum neutralizing activity of mRNA-1273 against SARS-CoV-2 variants. bioRxiv.

[61] Fakih S., Kamilli A., Jödicke-Braas B. CureVac Provides Update on Phase 2b/3 Trial of First-Generation COVID-19 Vaccine Candidate, CVnCoV. https://www.curevac.com/en/2021/06/16/curevac-provides-update-on-phase-2b-3-trial-of-first-generation-covid-19-vaccine-candidate-cvncov/.

[62] Fakih S., Kamilli A., Jödicke-Braas B. CureVac Final Data from Phase 2b/3 Trial of First-Generation COVID-19 Vaccine Candidate, CVnCoV, Demonstrates Protection in Age Group of 18 to 60. https://www.curevac.com/en/2021/06/30/curevac-final-data-from-phase-2b-3-trial-of-first-generation-covid-19-vaccine-candidate-cvncov-demonstrates-protection-in-age-group-of-18-to-60/.

[63] Zhang N.N., Li X.F., Deng Y.Q., Zhao H., Huang Y.J., Yang G., Huang W.J., Gao P., Zhou C., Zhang R.R. (2020). A Thermostable mRNA Vaccine against COVID-19. Cell.

[64] Holdings P.T. Providence Therapeutics Announces Very Favorable Interim Phase 1 Trial Data for PTX-COVID19-B, its mRNA Vaccine against COVID-19. https://www.providencetherapeutics.com/providence-therapeutics-announces-very-favorable-interim-phase-1-trial-data-for-ptx-covid19-b-its-mrna-vaccine-against-covid-19.

[65] Liu J., Budylowski P., Samson R., Griffin B.D., Babuadze G., Rathod B., Colwill K., Abioye J.A., Schwartz J.A., Law R. (2021). Preclinical evaluation of a SARS-CoV-2 mRNA vaccine PTX-COVID19-B. bioRxiv.

[66] Hatmal M.M., Al-Hatamleh M.A.I., Olaimat A.N., Hatmal M., Alhaj-Qasem D.M., Olaimat T.M., Mohamud R. (2021). Side Effects and Perceptions Following COVID-19 Vaccination in Jordan: A Randomized, Cross-Sectional Study Implementing Machine Learning for Predicting Severity of Side Effects. Vaccines.

[67] Abu-Raddad L.J., Chemaitelly H., Butt A.A., National Study Group for C.-V. (2021). Effectiveness of the BNT162b2 COVID-19 Vaccine against the B.1.1.7 and B.1.351 Variants. N. Engl. J. Med..

[68] Lubell M. Healthcare & Pharmaceuticals: Pfizer Says COVID vaccine Is Highly effective against Delta Variant. https://www.pharmalive.com/pfizer-says-covid-vaccine-highly-effective-against-delta-variant/.

[69] U.S. Food and Drug Administration COVID-19 Vaccines. https://www.fda.gov/emergency-preparedness-and-response/coronavirus-disease-2019-covid-19/covid-19-vaccines.

[70] U.S. Food and Drug Administration Fact Sheet for Recipients and Caregivers: Emergency Use Authorization (Eua) of the Pfizer-Biontech COVID-19 Vaccine to Prevent Coronavirus Disease 2019 (COVID-19) In Individuals 12 Years of Age and Older. https://www.fda.gov/media/144414/download.

[71] Katella K. Comparing the COVID-19 Vaccines: How Are They Different?. https://www.yalemedicine.org/news/covid-19-vaccine-comparison.

[72] Kariko K., Muramatsu H., Welsh F.A., Ludwig J., Kato H., Akira S., Weissman D. (2008). Incorporation of pseudouridine into mRNA yields superior nonimmunogenic vector with increased translational capacity and biological stability. Mol. Ther..

[73] Wrapp D., Wang N., Corbett K.S., Goldsmith J.A., Hsieh C.L., Abiona O., Graham B.S., McLellan J.S. (2020). Cryo-EM structure of the 2019-nCoV spike in the prefusion conformation. Science.

[74] Ravell J.C. A Simple Breakdown of the Ingredients in the COVID Vaccines. https://www.hackensackmeridianhealth.org/HealthU/2021/01/11/a-simple-breakdown-of-the-ingredients-in-the-covid-vaccines/.

[75] Lawton G. Everything you Need to Know about the Pfizer/BioNTech COVID-19 Vaccine. https://www.newscientist.com/article/2261805-everything-you-need-to-know-about-the-pfizer-biontech-covid-19-vaccine/.

[76] Stowe J., Andrews N., Gower C., Gallagher E., Utsi L., Simmons R., Thelwall S., Tessier E., Groves N., Dabrera G. (2021). Effectiveness of COVID-19 Vaccines against Hospital Admission with the Delta (B.1.617.2) Variant. https://media.tghn.org/articles/Effectiveness_of_COVID-19_vaccines_against_hospital_admission_with_the_Delta_B._G6gnnqJ.pdf.

[77] Bernal J.L., Andrews N., Gower C., Gallagher E., Simmons R., Thelwall S., Stowe J., Tessier E., Groves N., Dabrera G. (2021). Effectiveness of COVID-19 vaccines against the B.1.617.2 variant. medRvix.

[78] Thomas S.J., Moreira E.D., Kitchin N., Absalon J., Gurtman A., Lockhart S., Perez J.L., Marc G.P., Polack F.P., Zerbini C. (2021). Six Month Safety and Efficacy of the BNT162b2 mRNA COVID-19 Vaccine. medRvix.

[79] Bernal J.L., Andrews N., Gower C., Gallagher E., Simmons R., Thelwall S., Stowe J., Tessier E., Groves N., Dabrera G. (2021). Effectiveness of COVID-19 Vaccines against the B.1.617.2 (Delta) Variant. N. Engl. J. Med..

[80] Thompson M.G., Burgess J.L., Naleway A.L., Tyner H.L., Yoon S.K., Meece J., Olsho L.E., Caban-Martinez A.J., Fowlkes A., Lutrick K. (2021). Interim Estimates of Vaccine Effectiveness of BNT162b2 and mRNA-1273 COVID-19 Vaccines in Preventing SARS-CoV-2 Infection Among Health Care Personnel, First Responders, and Other Essential and Frontline Workers—Eight U.S. Locations, December 2020–March 2021. MMWR Morb. Mortal. Wkly. Rep..

[81] Moderna Moderna Provides Clinical and Supply Updates on COVID-19 Vaccine Program ahead of 2nd Annual Vaccines Day. https://www.businesswire.com/news/home/20210413006131/en/.

[82] Baden L.R., El Sahly H.M., Essink B., Kotloff K., Frey S., Novak R., Diemert D., Spector S.A., Rouphael N., Creech C.B. (2021). Efficacy and safety of the mRNA-1273 SARS-CoV-2 vaccine. N. Engl. J. Med..

[83] Wu K., Werner A.P., Koch M., Choi A., Narayanan E., Stewart-Jones G.B.E., Colpitts T., Bennett H., Boyoglu-Barnum S., Shi W. (2021). Serum Neutralizing Activity Elicited by mRNA-1273 Vaccine. N. Engl. J. Med..

[84] Moderna US, IFact Sheet for Recipients and Caregivers: Emergency Use Authorization (EUA) of the Moderna COVID-19 Vaccine to Prevent Coronavirus Disease 2019 (COVID-19) in Individuals 18 Years of Age and Older. https://www.fda.gov/media/144638/download.

[85] Modena Moderna Provides a Clinical Update on the Neutralizing Activity of its COVID-19 Vaccine on Emerging Variants Including the Delta Variant First Identified in India. https://www.businesswire.com/news/home/20210629005708/en/.

[86] Modena Moderna Announces First Participants Dosed in Study Evaluating COVID-19 Booster Vaccine Candidates. https://investors.modernatx.com/news-releases/news-release-details/moderna-announces-first-participants-dosed-study-evaluating.

[87] Oncotelic Therapeutics Oncotelic Therapeutics, Inc. Announces Positive Top Line Data for Arti-19 Clinical Trial Evaluating Pulmoheal™ versus COVID-19. https://www.globenewswire.com/en/news-re-lease/2021/04/20/2213564/0/en/ONCOTELIC-THERAPEUTICS-INC-ANNOUNCES-POSITIVE-TOP-LINE-DATA-FOR-ARTI-19-CLINICAL-TRIAL-EVALUATING-PULMOHEAL-VERSUS-COVID-19.html.

[88] AIM ImmunoTech AIM ImmunoTech Announces Positive Safety Data in Second Cohort of Phase 1 Clinical Study Investigating Intranasal Administration of Ampligen as a Potential Prophylaxis or Treatment for COVID-19 and Other Respiratory Viral Diseases. https://www.biospace.com/article/releases/aim-immunotech-announces-positive-safety-data-in-second-cohort-of-phase-1-clinical-study-investigating-intranasal-administration-of-ampligen-as-a-potential-prophylaxis-or-treatment-for-covid-19-and-other-respiratory-viral-diseases/.

[89] AIM ImmunoTech ImmunoTech Announces Positive Safety Data in First Cohort of Phase 1 Clinical Study Investigating Intranasal Administration of Ampligen as a Potential Prophylaxis or Treatment for COVID-19 and Other Respiratory Viral Diseases. https://www.biospace.com/article/releases/aim-immunotech-announces-positive-safty-data-in-first-cohort-of-phase-1-clinical-study-investigating-intranasal-administration-of-ampligen-as-a-potential-prophylaxis-or-treatment-for-covid-19-and-other-respiratory-viral-diseases/.

[90] Bhattacharyya P., Biswas S.C. (2020). Small Non-coding RNAs: Do They Encode Answers for Controlling SARS-CoV-2 in the Future?. Front. Microbiol..

[91] Mateon Therapeutics Mateon Achieves Milestone in its Development of OT-101, a Phase 3 Clinical Drug Candidate, Against COVID-19. https://www.globenewswire.com/en/news-release/2020/04/06/2012085/10132/en/Mateon-Achieves-Milestone-in-its-Development-of-OT-101-a-Phase-3-Clinical-Drug-Candidate-Against-COVID-19.html.

[92] Boumaza A., Gay L., Mezouar S., Bestion E., Diallo A.B., Michel M., Desnues B., Raoult D., La Scola B., Halfon P. (2021). Monocytes and Macrophages, Targets of Severe Acute Respiratory Syndrome Coronavirus 2: The Clue for Coronavirus Disease 2019 Immunoparalysis. J. Infect. Dis..

[93] Carvacho I., Piesche M. (2021). RGD-binding integrins and TGF-beta in SARS-CoV-2 infections—Novel targets to treat COVID-19 patients?. Clin. Transl. Immunol..

[94] Uckun F.M., Qazi S., Hwang L., Trieu V.N. (2019). Recurrent or Refractory High-Grade Gliomas Treated by Convection-Enhanced Delivery of a TGFbeta2-Targeting RNA Therapeutic: A Post-Hoc Analysis with Long-Term Follow-Up. Cancers.

[95] Mateon Therapeutics Mateon Report Positive Results for Multiple COVID-19 Drug CandidatesOT-101 and Two Additional Candidates Demonstrated Viral Inhibition Activity against Coronavirus. https://www.biospace.com/article/releases/mateon-report-positive-results-for-multiple-covid-19-drug-candidatesot-101-and-two-additional-candidates-demonstrated-viral-inhibition-activity-against-coronavirus/.

[96] Chan J.F., Yao Y., Yeung M.L., Deng W., Bao L., Jia L., Li F., Xiao C., Gao H., Yu P. (2015). Treatment with Lopinavir/Ritonavir or Interferon-beta1b Improves Outcome of MERS-CoV Infection in a Nonhuman Primate Model of Common Marmoset. J. Infect. Dis..

[97] Prokunina-Olsson L., Alphonse N., Dickenson R.E., Durbin J.E., Glenn J.S., Hartmann R., Kotenko S.V., Lazear H.M., O’Brien T.R., Odendall C. (2020). COVID-19 and emerging viral infections: The case for interferon lambda. J. Exp. Med..

[98] Simabuco F.M., Tamura R.E., Pavan I.C.B., Morale M.G., Ventura A.M. (2020). Molecular mechanisms and pharmacological interventions in the replication cycle of human coronaviruses. Genet. Mol. Biol..

[99] AIM ImmunoTech Ampligen’s Update in COVID-19, ME/CFS, COVID-19 Induced Fatigue and Immuno-Oncology Alferon Manufacturing Update. https://d2ghdaxqb194v2.cloudfront.net/2265/181798.pdf.

[100] Barnard D.L., Day C.W., Bailey K., Heiner M., Montgomery R., Lauridsen L., Chan P.K., Sidwell R.W. (2006). Evaluation of immunomodulators, interferons and known in vitro SARS-coV inhibitors for inhibition of SARS-coV replication in BALB/c mice. Antivir. Chem. Chemother..

[101] Day C.W., Baric R., Cai S.X., Frieman M., Kumaki Y., Morrey J.D., Smee D.F., Barnard D.L. (2009). A new mouse-adapted strain of SARS-CoV as a lethal model for evaluating antiviral agents in vitro and in vivo. Virology.

[102] Frieman M., Heise M., Baric R. (2008). SARS coronavirus and innate immunity. Virus Res..

[103] Sadler A.J., Williams B.R. (2008). Interferon-inducible antiviral effectors. Nat. Rev. Immunol..

[104] Soppe J.A., Lebbink R.J. (2017). Antiviral goes viral: Harnessing CRISPR/Cas9 to combat viruses in humans. Trends Microbiol..

[105] INOVIO Pharmaceuticals INOVIO Announces Positive Interim Phase 1 Data for INO-4800 Vaccine for COVID-19. http://ir.inovio.com/news-releases/news-releases-details/2020/INOVIO-Announces-Positive-Interim-Phase-1-Data-For-INO-4800-Vaccine-for-COVID-19/default.aspx.

[106] Fomsgaard A. The Statens Serum Institut is Developing a New Promising. https://www.ssi.dk/aktuelt/nyheder/2020/statens-serum-institut-udvikler-en-ny-lovende-vaccine-mod-covid-19.

[107] Clinical Trials Arena University of Cambridge to Trial COVID-19 Vaccine Candidate. https://www.clinicaltrialsarena.com/news/cambridge-uni-covid-vaccine/.

[108] Rego G.N.A., Nucci M.P., Alves A.H., Oliveira F.A., Marti L.C., Nucci L.P., Mamani J.B., Gamarra L.F. (2020). Current Clinical Trials Protocols and the Global Effort for Immunization against SARS-CoV-2. Vaccines.

[109] Zydus Cadila Zydus Cadila Announces Completion of Dosing in Phase I Clinical Trial of ZyCoV-D. https://pipelinereview.com/index.php/2020080575496/Vaccines/Zydus-Cadila-Announces-Completion-of-Dosing-in-Phase-I-Clinical-Trial-of-ZyCoV-D.html.

[110] Kumar V.M., Pandi-Perumal S.R., Trakht I., Thyagarajan S.P. (2021). Strategy for COVID-19 vaccination in India: The country with the second highest population and number of cases. Npj Vaccines.

[111] Precision Vaccinations ZyCoV-D COVID-19 Vaccine. https://www.precisionvaccinations.com/vaccines/zycov-d-covid-19-vaccine.

[112] Kaur S.P., Gupta V. (2020). COVID-19 Vaccine: A comprehensive status report. Virus. Res..

[113] Zydus Cadila ZyCoV-D: Zydus Cadila to Begin Phase II Human Trials of COVID-19 Vaccine from Tomorrow. https://www.indiatvnews.com/business/news-zycov-d-coronavirus-vaccine-zydus-cadila-phase-ii-clinical-trials-covid-19-vaccine-august-6-639700.

[114] Zydus Cadila (2021). Zydus Applies to the DCGI for EUA to Launch ZyCoV-D, the World’s First Plasmid DNA Vaccine for COVID-19. https://www.zyduscadila.com/public/pdf/pressrelease/ZyCoV_D_Press_Release_1_7_2021.pdf.

[115] Brisse M., Vrba S.M., Kirk N., Liang Y., Ly H. (2020). Emerging Concepts and Technologies in Vaccine Development. Front. Immunol..

[116] Rawat K., Kumari P., Saha L. (2021). COVID-19 vaccine: A recent update in pipeline vaccines, their design and development strategies. Eur. J. Pharmacol..

[117] Precision Vaccinations AG0301 COVID-19 Vaccine Description. https://www.precisionvaccinations.com/vaccines/ag0301-covid-19-vaccine.

[118] AnGes I. Study of COVID-19 DNA Vaccine (AG0301-COVID19). https://clinicaltrials.gov/ct2/show/NCT04463472.

[119] AnGes I. Phase II/III Study of COVID-19 DNA Vaccine (AG0302-COVID19). https://clinicaltrials.gov/ct2/show/NCT04655625.

[120] Pharmaceuticals I. Safety, Tolerability and Immunogenicity of INO-4800 for COVID-19 in Healthy Volunteers. https://clinicaltrials.gov/ct2/show/NCT04336410.

[121] Precision Vaccinations INO-4800 COVID-19 Vaccine. https://www.precisionvaccinations.com/vaccines/ino-4800-covid-19-vaccine.

[122] Smith T.R., Patel A., Ramos S., Elwood D., Zhu X., Yan J., Gary E.N., Walker S.N., Schultheis K., Purwar M. (2020). Immunogenicity of a DNA vaccine candidate for COVID-19. Nat. Commun..

[123] Patel A., Walters J., Reuschel E.L., Schultheis K., Parzych E., Gary E.N., Maricic I., Purwar M., Eblimit Z., Walker S.N. (2020). Intradermal-delivered DNA vaccine provides anamnestic protection in a rhesus macaque SARS-CoV-2 challenge model. BioRxiv.

[124] INOVIO Reports Positive Interim Phase I Data for COVID-19 DNA Vaccine, Joins “Warp Speed” Primate Study. https://www.genengnews.com/news/inovio-reports-positive-interim-phase-i-data-for-covid-19-dna-vaccine-joins-warp-speed-primate-study/.

[125] Tebas P., Yang S., Boyer J.D., Reuschel E.L., Patel A., Christensen-Quick A., Andrade V.M., Morrow M.P., Kraynyak K., Agnes J. (2021). Safety and immunogenicity of INO-4800 DNA vaccine against SARS-CoV-2: A preliminary report of an open-label, Phase 1 clinical trial. EClinicalMedicine.

[126] Andrade V.M., Christensen-Quick A., Agnes J., Tur J., Reed C., Kalia R., Marrero I., Elwood D., Schultheis K., Purwar M. (2021). INO-4800 DNA vaccine induces neutralizing antibodies and T cell Activity against global SARS-CoV-2 variants. bioRxiv.

[127] Mammen M.P., Tebas P., Agnes J., Giffear M., Kraynyak K.A., Blackwood E., Amante D., Reuschel E.L., Purwar M., Christensen-Quick A. (2021). Safety and immunogenicity of INO-4800 DNA vaccine against SARS-CoV-2: A preliminary report of a randomized, blinded, placebo-controlled, Phase 2 clinical trial in adults at high risk of viral exposure. medRxiv.

[128] INOVIO INOVIO Announces Initiation of Phase 2 Segment of its Phase 2/3 Clinical Trial for its COVID-19 DNA Vaccine Candidate, INO-4800. https://ir.inovio.com/news-releases/news-releases-details/2020/INOVIO-Announces-Initiation-of-Phase-2-Segment-of-its-Phase-23-Clinical-Trial-for-its-COVID-19-DNA-Vaccine-Candidate-INO-4800-Trial-Will-Be-Funded-by-the-U.S.-Department-of-Defense/default.aspx.

[129] INOVIO INOVIO’s Pan-COVID-19 Vaccine Candidate (INO4802) Induces Broad Immunity Against Major Viral Variants in Preclinical Studies. https://ir.inovio.com/news-releases/news-releases-details/2021/INOVIOs-Pan-COVID-19-Vaccine-Candidate-INO-4802-Induces-Broad-Immunity-Against-Major-Viral-Variants-in-Preclinical-Studies/default.aspx.

[130] Ma H., Zeng W., He H., Zhao D., Jiang D., Zhou P., Cheng L., Li Y., Ma X., Jin T. (2020). Serum IgA, IgM, and IgG responses in COVID-19. Cell. Mol. Immunol..

[131] Han H., Ma Q., Li C., Liu R., Zhao L., Wang W., Zhang P., Liu X., Gao G., Liu F. (2020). Profiling serum cytokines in COVID-19 patients reveals IL-6 and IL-10 are disease severity predictors. Emerg. Microbes Infect..

[132] Liu B.M., Martins T.B., Peterson L.K., Hill H.R. (2021). Clinical significance of measuring serum cytokine levels as inflammatory biomarkers in adult and pediatric COVID-19 cases: A review. Cytokine.

[133] McElvaney O.J., McEvoy N.L., McElvaney O.F., Carroll T.P., Murphy M.P., Dunlea D.M., Ní Choileáin O., Clarke J., O’Connor E., Hogan G. (2020). Characterization of the inflammatory response to severe COVID-19 illness. Am. J. Respir. Crit. Care Med..

[134] Ragab D., Salah Eldin H., Taeimah M., Khattab R., Salem R. (2020). The COVID-19 cytokine storm; what we know so far. Front. Immunol..

[135] Panigrahy D., Gilligan M.M., Huang S., Gartung A., Cortés-Puch I., Sime P.J., Phipps R.P., Serhan C.N., Hammock B.D. (2020). Inflammation resolution: A dual-pronged approach to averting cytokine storms in COVID-19?. Cancer Metastasis Rev..

[136] Bhaskar S., Sinha A., Banach M., Mittoo S., Weissert R., Kass J.S., Rajagopal S., Pai A.R., Kutty S. (2020). Cytokine storm in COVID-19-immunopathological mechanisms, clinical considerations, and therapeutic approaches: The REPROGRAM consortium position paper. Front. Immunol..

[137] Chen G., Wu D., Guo W., Cao Y., Huang D., Wang H., Wang T., Zhang X., Chen H., Yu H. (2020). Clinical and immunological features of severe and moderate coronavirus disease 2019. J. Clin. Investig..

[138] Zhou F., Yu T., Du R., Fan G., Liu Y., Liu Z., Xiang J., Wang Y., Song B., Gu X. (2020). Clinical course and risk factors for mortality of adult inpatients with COVID-19 in Wuhan, China: A retrospective cohort study. Lancet.

[139] Hirano T., Murakami M. (2020). COVID-19: A new virus, but a familiar receptor and cytokine release syndrome. Immunity.

[140] Liu B., Li M., Zhou Z., Guan X., Xiang Y. (2020). Can we use interleukin-6 (IL-6) blockade for coronavirus disease 2019 (COVID-19)-induced cytokine release syndrome (CRS)?. J. Autoimmun..

[141] Chen Y., Liu Q., Guo D. (2020). Emerging coronaviruses: Genome structure, replication, and pathogenesis. J. Med. Virol..

[142] Challen R., Brooks-Pollock E., Read J.M., Dyson L., Tsaneva-Atanasova K., Danon L. (2021). Risk of mortality in patients infected with SARS-CoV-2 variant of concern 202012/1: Matched cohort study. BMJ.

[143] Zhou D., Dejnirattisai W., Supasa P., Liu C., Mentzer A.J., Ginn H.M., Zhao Y., Duyvesteyn H.M.E., Tuekprakhon A., Nutalai R. (2021). Evidence of escape of SARS-CoV-2 variant B.1.351 from natural and vaccine-induced sera. Cell.

[144] Mayo Clinic COVID-19 Variants: What’s the Concern?. https://www.mayoclinic.org/diseases-conditions/coronavirus/expert-answers/covid-variant/faq-20505779.

[145] Hodcroft E. Variant: 21A (Delta). https://covariants.org/variants/21A.Delta.

[146] WHO Tracking SARS-CoV-2 Variants. https://www.who.int/en/activities/tracking-SARS-CoV-2-variants/.

[147] Hagen A. How Dangerous Is the Delta Variant (B.1.617.2)?. https://asm.org/Articles/2021/July/How-Dangerous-is-the-Delta-Variant-B-1-617-2.

[148] Abbott B. Delta Variant Accounts for 83% of Known U.S. Covid-19 Cases. https://www.wsj.com/articles/delta-variant-accounts-for-83-of-known-covid-19-cases-11626807019.

[149] WHO Update on Omicron. https://www.who.int/news/item/28-11-2021-update-on-omicron.

[150] Geddes L. What Do We Know about the New B.1.1.529 Coronavirus Variant and Should We Be Worried?. https://www.gavi.org/vaccineswork/what-we-know-about-new-b11529-coronavirus-variant-so-far?gclid=Cj0KCQiAip-PBhDVARIsAPP2xc3Q_oSb2Vr7HHnELNZmKy2-vrIG_qeiugLkRy3k3mjodpSQfcg_T78aAiVVEALw_wcB.

[151] Sinha A. ‘IHU’ Variant of COVID-19 Explained: Few Cases, Limited Spread. https://indianexpress.com/article/explained/ihu-variant-few-cases-limited-spread-7706641/.

[152] Le T.K., Paris C., Khan K.S., Robson F., Ng W.-L., Rocchi P. (2021). Nucleic acid-based technologies targeting coronaviruses. Trends Biochem. Sci..

[153] Abbott T.R., Dhamdhere G., Liu Y., Lin X., Goudy L., Zeng L., Chemparathy A., Chmura S., Heaton N.S., Debs R. (2020). Development of CRISPR as an antiviral strategy to combat SARS-CoV-2 and influenza. Cell.

[154] Sridharan K., Gogtay N.J. (2016). Therapeutic nucleic acids: Current clinical status. Br. J. Clin. Pharmacol..

[155] Gewirtz A.M., Sokol D.L., Ratajczak M.Z. (1998). Nucleic acid therapeutics: State of the art and future prospects. Blood.

[156] Yan Z.P., Yang M., Lai C.L. (2021). COVID-19 Vaccines: A Review of the Safety and Efficacy of Current Clinical Trials. Pharmaceuticals.

[157] Dash P., Mohapatra S., Ghosh S., Nayak B. (2020). A Scoping Insight on Potential Prophylactics, Vaccines and Therapeutic Weaponry for the Ongoing Novel Coronavirus (COVID-19) Pandemic- A Comprehensive Review. Front. Pharmacol..

[158] Flanagan K.L., Best E., Crawford N.W., Giles M., Koirala A., Macartney K., Russell F., Teh B.W., Wen S.C. (2020). Progress and Pitfalls in the Quest for Effective SARS-CoV-2 (COVID-19) Vaccines. Front. Immunol..

[159] Berber B., Aydin C., Kocabas F., Guney-Esken G., Yilancioglu K., Karadag-Alpaslan M., Caliseki M., Yuce M., Demir S., Tastan C. (2021). Gene editing and RNAi approaches for COVID-19 diagnostics and therapeutics. Gene Ther..

[160] Kim D., Rossi J. (2008). RNAi mechanisms and applications. BioTechniques.

[161] Lee Y., Hur I., Park S.-Y., Kim Y.-K., Suh M.R., Kim V.N. (2006). The role of PACT in the RNA silencing pathway. EMBO J..

[162] Settleman J., Sawyers C.L., Hunter T. (2018). Challenges in validating candidate therapeutic targets in cancer. eLife.

[163] Deng Y., Wang C.C., Choy K.W., Du Q., Chen J., Wang Q., Li L., Chung T.K.H., Tang T. (2014). Therapeutic potentials of gene silencing by RNA interference: Principles, challenges, and new strategies. Gene.

[164] Elbashir S.M., Harborth J., Lendeckel W., Yalcin A., Weber K., Tuschl T. (2001). Duplexes of 21-nucleotide RNAs mediate RNA interference in cultured mammalian cells. Nature.

[165] Medeiros I.G., Khayat A.S., Stransky B., Santos S., Assumpção P., de Souza J.E.S. (2021). A small interfering RNA (siRNA) database for SARS-CoV-2. Sci. Rep..

[166] Chen W., Feng P., Liu K., Wu M., Lin H. (2020). Computational identification of small interfering RNA targets in SARS-CoV-2. Virol. Sin..

[167] Idris A., Davis A., Supramaniam A., Acharya D., Kelly G., Tayyar Y., West N., Zhang P., McMillan C.L., Soemardy C. (2021). A SARS-CoV-2 targeted siRNA-nanoparticle therapy for COVID-19. Mol. Ther..

[168] Niktab I., Haghparast M., Beigi M.-H., Megraw T.L., Kiani A., Ghaedi K. (2021). Design of advanced siRNA therapeutics for the treatment of COVID-19. Meta Gene.

[169] Liu C., Zhou Q., Li Y., Garner L.V., Watkins S.P., Carter L.J., Smoot J., Gregg A.C., Daniels A.D., Jervey S. (2020). Research and development on therapeutic agents and vaccines for COVID-19 and related human coronavirus diseases. ACS Cent. Sci..

[170] Ullah A., Qazi J., Rahman L., Kanaras A.G., Khan W.S., Hussain I., Rehman A. (2020). Nanoparticles-assisted delivery of antiviral-siRNA as inhalable treatment for human respiratory viruses: A candidate approach against SARS-COV-2. Nano Sel..

[171] Farr R., Rootes C., Rowntree L., Nguyen T., Hensen L., Kedzierski L., Cheng A., Kedzierska K., Au G., Marsh G. (2021). Altered microRNA expression in COVID-19 patients enables identification of SARS-CoV-2 infection. PLoS Pathog..

[172] Friedman R.C., Farh K.K.-H., Burge C.B., Bartel D.P. (2009). Most mammalian mRNAs are conserved targets of microRNAs. Genome Res..

[173] Hum C., Loiselle J., Ahmed N., Shaw T.A., Toudic C., Pezacki J.P. (2021). MicroRNA mimics or inhibitors as antiviral therapeutic approaches against COVID-19. Drugs.

[174] Balmeh N., Mahmoudi S., Mohammadi N., Karabedianhajiabadi A. (2020). Predicted therapeutic targets for COVID-19 disease by inhibiting SARS-CoV-2 and its related receptors. Inform. Med. Unlocked.

[175] Narozna M., Rubis B. (2021). Anti-SARS-CoV-2 Strategies and the Potential Role of miRNA in the Assessment of COVID-19 Morbidity, Recurrence, and Therapy. Int. J. Mol. Sci..

[176] Han J., Lee Y., Yeom K.-H., Nam J.-W., Heo I., Rhee J.-K., Sohn S.Y., Cho Y., Zhang B.-T., Kim V.N. (2006). Molecular basis for the recognition of primary microRNAs by the Drosha-DGCR8 complex. Cell.

[177] Catalanotto C., Cogoni C., Zardo G. (2016). MicroRNA in control of gene expression: An overview of nuclear functions. Int. J. Mol. Sci..

[178] Hu J., Stojanovic J., Yasamineh S., Yasamineh P., Karuppannan S.K., Hussain Dowlath M.J., Serati-Nouri H. (2021). The potential use of microRNAs as a therapeutic strategy for SARS-CoV-2 infection. Arch. Virol..

[179] Gallant-Behm C.L., Piper J., Lynch J.M., Seto A.G., Hong S.J., Mustoe T.A., Maari C., Pestano L.A., Dalby C.M., Jackson A.L. (2019). A microRNA-29 mimic (Remlarsen) represses extracellular matrix expression and fibroplasia in the skin. J. Investig. Dermatol..

[180] Seto A.G., Beatty X., Lynch J.M., Hermreck M., Tetzlaff M., Duvic M., Jackson A.L. (2018). Cobomarsen, an oligonucleotide inhibitor of miR-155, co-ordinately regulates multiple survival pathways to reduce cellular proliferation and survival in cutaneous T-cell lymphoma. Br. J. Haematol..

[181] Lam J.K.W., Chow M.Y.T., Zhang Y., Leung S.W.S. (2015). siRNA Versus miRNA as therapeutics for gene silencing. Mol. Ther. Nucleic Acids.

[182] Iorio M.V., Croce C.M. (2012). MicroRNA dysregulation in cancer: Diagnostics, monitoring and therapeutics. A comprehensive review. EMBO Mol. Med..

[183] Hosseini Rad Sm A., McLellan A.D. (2020). Implications of SARS-CoV-2 mutations for genomic RNA structure and host microRNA targeting. Int. J. Mol. Sci..

[184] Maitra A., Sarkar M.C., Raheja H., Biswas N.K., Chakraborti S., Singh A.K., Ghosh S., Sarkar S., Patra S., Mondal R.K. (2020). Mutations in SARS-CoV-2 viral RNA identified in Eastern India: Possible implications for the ongoing outbreak in India and impact on viral structure and host susceptibility. J. Biosci..

[185] Lu D., Chatterjee S., Xiao K., Riedel I., Wang Y., Foo R., Bär C., Thum T. (2020). MicroRNAs targeting the SARS-CoV-2 entry receptor ACE2 in cardiomyocytes. J. Mol. Cell. Cardiol..

[186] Matarese A., Gambardella J., Sardu C., Santulli G. (2020). miR-98 regulates TMPRSS2 expression in human endothelial cells: Key implications for COVID-19. Biomedicines.

[187] Kaur T., Kapila S., Kapila R., Kumar S., Upadhyay D., Kaur M., Sharma C. (2021). Tmprss2 specific miRNAs as promising regulators for SARS-CoV-2 entry checkpoint. Virus Res..

[188] Sardar R., Satish D., Birla S., Gupta D. (2020). Dataset of mutational analysis, miRNAs targeting SARS-CoV-2 genes and host gene expression in SARS-CoV and SARS-CoV-2 infections. Data Brief..

[189] Chow J.T.-S., Salmena L. (2020). Prediction and analysis of SARS-CoV-2-targeting microRNA in human lung epithelium. Genes.

[190] Ge J., Li J., Na S., Wang P., Zhao G., Zhang X. (2019). miR-548c-5p inhibits colorectal cancer cell proliferation by targeting PGK1. J. Cell. Physiol..

[191] Fulzele S., Sahay B., Yusufu I., Lee T.J., Sharma A., Kolhe R., Isales C.M. (2020). COVID-19 virulence in aged patients might be impacted by the host cellular microRNAs abundance/profile. Aging Dis..

[192] El-Nabi S.H., Elhiti M., El-Sheekh M. (2020). A new approach for COVID-19 treatment by micro-RNA. Med. Hypotheses.

[193] Khan M.A.-A.-K., Sany M.R.U., Islam M.S., Islam A.B.M.M.K. (2020). Epigenetic regulator miRNA pattern differences among SARS-CoV, SARS-CoV-2, and SARS-CoV-2 world-wide isolates delineated the mystery behind the epic pathogenicity and distinct clinical characteristics of pandemic COVID-19. Front. Genet..

[194] Saçar Demirci M.D., Adan A. (2020). Computational analysis of microRNA-mediated interactions in SARS-CoV-2 infection. PeerJ.

[195] Ahmed S.S.S.J., Paramasivam P., Raj K., Kumar V., Murugesan R., Ramakrishnan V. (2020). Regulatory cross talk between SARS-CoV-2 receptor binding and replication machinery in the human host. Front. Physiol..

[196] Khan A.T.-A., Khalid Z., Zahid H., Yousaf M.A., Shakoori A.R. (2020). A computational and bioinformatic analysis of ACE2: An elucidation of its dual role in COVID-19 pathology and finding its associated partners as potential therapeutic targets. J. Biomol. Struct. Dyn..

[197] Zhang H., Rostami M.R., Leopold P.L., Mezey J.G., O’Beirne S.L., Strulovici-Barel Y., Crystal R.G. (2020). Expression of the SARS-CoV-2 ACE2 receptor in the human airway epithelium. Am. J. Respir. Crit. Care Med..

[198] Nersisyan S., Shkurnikov M., Turchinovich A., Knyazev E., Tonevitsky A. (2020). Integrative analysis of miRNA and mRNA sequencing data reveals potential regulatory mechanisms of ACE2 and TMPRSS2. PLoS ONE.

[199] Nersisyan S.A., Shkurnikov M.Y., Osipyants A.I., Vechorko V.I. (2020). Role of ACE2/TMPRSS2 genes regulation by intestinal microRNA isoforms in the COVID-19 pathogenesis. Bull. Russ. State Med. Univ..

[200] Arisan E.D., Dart A., Grant G.H., Arisan S., Cuhadaroglu S., Lange S., Uysal-Onganer P. (2020). The prediction of miRNAs in SARS-CoV-2 genomes: Hsa-miR databases identify 7 key miRs linked to host responses and virus pathogenicity-related KEGG pathways significant for comorbidities. Viruses.

[201] Chen L., Zhong L. (2020). Genomics functional analysis and drug screening of SARS-CoV-2. Genes Dis..

[202] Sardar R., Satish D., Birla S., Gupta D. (2020). Integrative analyses of SARS-CoV-2 genomes from different geographical locations reveal unique features potentially consequential to host-virus interaction, pathogenesis and clues for novel therapies. Heliyon.

[203] Mukhopadhyay D., Mussa B.M. (2020). Identification of novel hypothalamic microRNAs as promising therapeutics for SARS-CoV-2 by regulating ACE2 and TMPRSS2 expression: An in silico analysis. Brain Sci..

[204] Taz T.A., Ahmed K., Paul B.K., Kawsar M., Aktar N., Mahmud S.M.H., Moni M.A. (2021). Network-based identification genetic effect of SARS-CoV-2 infections to Idiopathic pulmonary fibrosis (IPF) patients. Brief. Bioinform..

[205] Vastrad B., Vastrad C., Tengli A. (2020). Identification of potential mRNA panels for severe acute respiratory syndrome coronavirus 2 (COVID-19) diagnosis and treatment using microarray dataset and bioinformatics methods. 3 Biotech.

[206] Marchi R., Sugita B., Centa A., Fonseca A.S., Bortoletto S., Fiorentin K., Ferreira S., Cavalli L.R. (2021). The role of microRNAs in modulating SARS-CoV-2 infection in human cells: A systematic review. Infection, genetics and evolution. J. Mol. Epidemiol. Evol. Genet. Infect. Dis..

[207] Zhang S., Amahong K., Sun X., Lian X., Liu J., Sun H., Lou Y., Zhu F., Qiu Y. (2021). The miRNA: A small but powerful RNA for COVID-19. Brief. Bioinform..

[208] Fani M., Zandi M., Ebrahimi S., Soltani S., Abbasi S. (2021). The role of miRNAs in COVID-19 disease. Future Virol..

[209] Gambardella J., Sardu C., Morelli M.B., Messina V., Castellanos V., Marfella R., Maggi P., Paolisso G., Wang X., Santulli G. (2020). Exosomal microRNAs drive thrombosis in COVID-19. medRxiv.

[210] Gallagher T.M., Buchmeier M.J. (2001). Coronavirus spike proteins in viral entry and pathogenesis. Virology.

[211] Alam T., Lipovich L. (2021). miRCOVID-19: Potential targets of human miRNAs in SARS-CoV-2 for RNA-based drug discovery. Non-Coding RNA.

[212] Wang Y., Zhang S., Li F., Zhou Y., Zhang Y., Wang Z., Zhang R., Zhu J., Ren Y., Tan Y. (2020). Therapeutic target database 2020: Enriched resource for facilitating research and early development of targeted therapeutics. Nucleic Acids Res..

[213] Mann M., Wright P.R., Backofen R. (2017). IntaRNA 2.0: Enhanced and customizable prediction of RNA-RNA interactions. Nucleic Acids Res..

[214] Ritchie W., Flamant S., Rasko J.E.J. (2010). mimiRNA: A microRNA expression profiler and classification resource designed to identify functional correlations between microRNAs and their targets. Bioinformatics.

[215] Dai X., Zhao P.X. (2011). psRNATarget: A plant small RNA target analysis server. Nucleic Acids Res..

[216] Betel D., Wilson M., Gabow A., Marks D.S., Sander C. (2008). The microRNA.org resource: Targets and expression. Nucleic Acids Res..

[217] Chou C.-H., Shrestha S., Yang C.-D., Chang N.-W., Lin Y.-L., Liao K.-W., Huang W.-C., Sun T.-H., Tu S.-J., Lee W.-H. (2018). miRTarBase update 2018: A resource for experimentally validated microRNA-target interactions. Nucleic Acids Res..

[218] Huang H.-Y., Lin Y.-C.-D., Li J., Huang K.-Y., Shrestha S., Hong H.-C., Tang Y., Chen Y.-G., Jin C.-N., Yu Y. (2020). miRTarBase 2020: Updates to the experimentally validated microRNA-target interaction database. Nucleic Acids Res..

[219] Pardi N., Hogan M.J., Porter F.W., Weissman D. (2018). mRNA vaccines—a new era in vaccinology. Nat. Rev. Drug Discov..

[220] Yi C., Yi Y., Li J. (2020). mRNA vaccines: Possible tools to combat SARS-CoV-2. Virol. Sin..

[221] McNamara M.A., Nair S.K., Holl E.K. (2015). RNA-based vaccines in cancer immunotherapy. J. Immunol. Res..

[222] Rosenberg Y., Sack M., Montefiori D., Labranche C., Lewis M., Urban L., Mao L., Fischer R., Jiang X. (2015). Pharmacokinetics and immunogenicity of broadly neutralizing HIV monoclonal antibodies in macaques. PLoS ONE.

[223] Kamboj M., Sepkowitz K.A. (2007). Risk of transmission associated with live attenuated vaccines given to healthy persons caring for or residing with an immunocompromised patient. Infect. Control. Hosp. Epidemiol..

[224] Lim B., Lee K. (2015). Stability of the osmoregulated promoter-derived *prop* mRNA is posttranscriptionally regulated by RNase III in *Escherichia coli*. J. Bacteriol..

[225] Pardi N., Weissman D. (2017). Nucleoside modified mRNA vaccines for infectious diseases. Methods Mol. Biol..

[226] Schlake T., Thess A., Thran M., Jordan I. (2019). mRNA as novel technology for passive immunotherapy. Cell. Mol. Life Sci..

[227] Jackson N.A.C., Kester K.E., Casimiro D., Gurunathan S., DeRosa F. (2020). The promise of mRNA vaccines: A biotech and industrial perspective. Npj Vaccines.

[228] Wang F., Kream R.M., Stefano G.B. (2020). An evidence based perspective on mRNA-SARS-CoV-2 vaccine development. Med. Sci. Monit. Int. Med. J. Exp. Clin. Res..

[229] Crommelin D.J.A., Anchordoquy T.J., Volkin D.B., Jiskoot W., Mastrobattista E. (2021). Addressing the cold reality of mRNA vaccine stability. J. Pharm. Sci..

[230] Reichmuth A.M., Oberli M.A., Jaklenec A., Langer R., Blankschtein D. (2016). mRNA vaccine delivery using lipid nanoparticles. Ther. Deliv..

[231] Vigerust D.J., Shepherd V.L. (2007). Virus glycosylation: Role in virulence and immune interactions. Trends Microbiol..

[232] Corbett K.S., Edwards D.K., Leist S.R., Abiona O.M., Boyoglu-Barnum S., Gillespie R.A., Himansu S., Schäfer A., Ziwawo C.T., DiPiazza A.T. (2020). SARS-CoV-2 mRNA vaccine design enabled by prototype pathogen preparedness. Nature.

[233] Xie X., Liu Y., Liu J., Zhang X., Zou J., Fontes-Garfias C.R., Xia H., Swanson K.A., Cutler M., Cooper D. (2021). Neutralization of SARS-CoV-2 spike 69/70 deletion, E484K and N501Y variants by BNT162b2 vaccine-elicited sera. Nat. Med..

[234] Skowronski D.M., Serres G.D. (2021). Safety and efficacy of the BNT162b2 mRNA COVID-19 vaccine. N. Engl. J. Med..

[235] Haas E.J., Angulo F.J., McLaughlin J.M., Anis E., Singer S.R., Khan F., Brooks N., Smaja M., Mircus G., Pan K. (2021). Impact and effectiveness of mRNA BNT162b2 vaccine against SARS-CoV-2 infections and COVID-19 cases, hospitalisations, and deaths following a nationwide vaccination campaign in Israel: An observational study using national surveillance data. Lancet.

[236] Simpson C.R., Shi T., Vasileiou E., Katikireddi S.V., Kerr S., Moore E., McCowan C., Agrawal U., Shah S.A., Ritchie L.D. (2021). First-dose ChAdOx1 and BNT162b2 COVID-19 vaccines and thrombocytopenic, thromboembolic and hemorrhagic events in Scotland. Nat. Med..

[237] Rauch S., Jasny E., Schmidt K.E., Petsch B. (2018). New vaccine technologies to combat outbreak situations. Front. Immunol..

[238] Rahimi H., Salehiabar M., Barsbay M., Ghaffarlou M., Kavetskyy T., Sharafi A., Davaran S., Chauhan S.C., Danafar H., Kaboli S. (2021). CRISPR systems for COVID-19 diagnosis. ACS Sens..

[239] Freije C.A., Myhrvold C., Boehm C.K., Lin A.E., Welch N.L., Carter A., Metsky H.C., Luo C.Y., Abudayyeh O.O., Gootenberg J.S. (2019). Programmable inhibition and detection of RNA viruses using Cas13. Mol. Cell.

[240] Barrey E., Burzio V., Dhorne-Pollet S., Eléouët J.-F., Delmas B. (2020). Think Different with RNA Therapy: Can Antisense Oligonucleotides Be Used to Inhibit Replication and Transcription of SARS-Cov-2?. Preprints.

[241] Gasparello J., Finotti A., Gambari R. (2021). Tackling the COVID-19 “cytokine storm” with microRNA mimics directly targeting the 3′UTR of pro-inflammatory mRNAs. Med. Hypotheses.

[242] Centa A., Fonseca A.S., da Silva Ferreira S.G., Azevedo M.L.V., Vaz de Paula C.B., Nagashima S., Machado-Souza C., dos Santos Miggiolaro A.F.R., Baena C.P., de Noronha L. (2020). Deregulated miRNA expression is associated with endothelial dysfunction in post-mortem lung biopsies of COVID-19 patients. Am. J. Physiol. Lung Cell. Mol. Physiol..

[243] Wicik Z., Eyileten C., Jakubik D., Simões S.N., Martins D.C., Pavão R., Siller-Matula J.M., Postula M. (2020). ACE2 interaction networks in COVID-19: A physiological framework for prediction of outcome in patients with cardiovascular risk factors. J. Clin. Med..

[244] Nersisyan S., Engibaryan N., Gorbonos A., Kirdey K., Makhonin A., Tonevitsky A. (2020). Potential role of cellular miRNAs in coronavirus-host interplay. PeerJ.

[245] Yamada K., Takizawa S., Ohgaku Y., Asami T., Furuya K., Yamamoto K., Takahashi F., Hamajima C., Inaba C., Endo K. (2020). MicroRNA 16-5p is upregulated in calorie-restricted mice and modulates inflammatory cytokines of macrophages. Gene.

[246] Ye E.-A., Liu L., Jiang Y., Jan J., Gaddipati S., Suvas S., Steinle J.J. (2016). miR-15a/16 reduces retinal leukostasis through decreased pro-inflammatory signaling. J. Neuroinflamm..

[247] Sun C.-M., Wu J., Zhang H., Shi G., Chen Z.-T. (2017). Circulating miR-125a but not miR-125b is decreased in active disease status and negatively correlates with disease severity as well as inflammatory cytokines in patients with Crohn’s disease. World J. Gastroenterol..

[248] Uludağ H., Parent K., Aliabadi H.M., Haddadi A. (2020). Prospects for RNAi therapy of COVID-19. Front. Bioeng. Biotechnol..

[249] Mai J., Virtue A., Maley E., Tran T., Yin Y., Meng S., Pansuria M., Jiang X., Wang H., Yang X.-F. (2012). MicroRNAs and other mechanisms regulate interleukin-17 cytokines and receptors. Front. Biosci. (Elite Ed.).

[250] Chen X., Zhou L., Peng N., Yu H., Li M., Cao Z., Lin Y., Wang X., Li Q., Wang J. (2017). MicroRNA-302a suppresses influenza A virus-stimulated interferon regulatory factor-5 expression and cytokine storm induction. J. Biol. Chem..

[251] Desjarlais M., Wirth M., Lahaie I., Ruknudin P., Hardy P., Rivard A., Chemtob S. (2020). Nutraceutical targeting of inflammation-modulating microRNAs in severe forms of COVID-19: A novel approach to prevent the cytokine storm. Front. Pharmacol..

[252] Gangemi S., Tonacci A. (2021). AntagomiRs: A novel therapeutic strategy for challenging COVID-19 cytokine storm. Cytokine Growth Factor Rev..

[253] Chen B.-B., Li Z.-H., Gao S. (2018). Circulating miR-146a/b correlates with inflammatory cytokines in COPD and could predict the risk of acute exacerbation COPD. Medicine.

[254] Liu Y., Guan H., Zhang J.-L., Zheng Z., Wang H.-T., Tao K., Han S.-C., Su L.-L., Hu D. (2018). Acute downregulation of miR-199a attenuates sepsis-induced acute lung injury by targeting SIRT1. Am. J. Physiol. Cell Physiol..

[255] Hu H.-L., Nie Z.-Q., Lu Y., Yang X., Song C., Chen H., Zhu S., Chen B.-B., Huang J., Geng S. (2017). Circulating miR-125b but not miR-125a correlates with acute exacerbations of chronic obstructive pulmonary disease and the expressions of inflammatory cytokines. Medicine.

[256] Garg A., Seeliger B., Derda A.A., Xiao K., Gietz A., Scherf K., Sonnenschein K., Pink I., Hoeper M.M., Welte T. (2021). Circulating cardiovascular microRNAs in critically ill COVID-19 patients. Eur. J. Heart Fail..

[257] Sabbatinelli J., Giuliani A., Matacchione G., Latini S., Laprovitera N., Pomponio G., Ferrarini A., Svegliati Baroni S., Pavani M., Moretti M. (2021). Decreased serum levels of the inflammaging marker miR-146a are associated with clinical non-response to tocilizumab in COVID-19 patients. Mech. Ageing Dev..

[258] Kalhori M.R., Saadatpour F., Arefian E., Soleimani M., Farzaei M.H., Aneva I.Y., Echeverria J. (2021). The Potential Therapeutic Effect of RNA Interference and Natural Products on COVID-19: A Review of the Coronaviruses Infection. Front. Pharmacol..

[259] Chen Z.-M., Fu J.-F., Shu Q., Chen Y.-H., Hua C.-Z., Li F.-B., Lin R., Tang L.-F., Wang T.-L., Wang W. (2020). Diagnosis and treatment recommendations for pediatric respiratory infection caused by the 2019 novel coronavirus. World J. Pediatr..

[260] Wang J., Zhu M., Ye L., Chen C., She J., Song Y. (2020). MiR-29b-3p promotes particulate matter-induced inflammatory responses by regulating the C1QTNF6/AMPK pathway. Aging.

[261] Plowman T., Lagos D. (2021). Non-Coding RNAs in COVID-19: Emerging Insights and Current Questions. Non-Coding RNA.

[262] OECD Access to COVID-19 Vaccines: Global Approaches in a Global Crisis. https://www.oecd.org/coronavirus/policy-responses/access-to-covid-19-vaccines-global-approaches-in-a-global-crisis-c6a18370/.

[263] Zhang C., Maruggi G., Shan H., Li J. (2019). Advances in mRNA Vaccines for Infectious Diseases. Front. Immunol..

[264] Kulkarni J.A., Witzigmann D., Thomson S.B., Chen S., Leavitt B.R., Cullis P.R., van der Meel R. (2021). The current landscape of nucleic acid therapeutics. Nat. Nanotechnol..

[265] Lee J., Arun Kumar S., Jhan Y.Y., Bishop C.J. (2018). Engineering DNA vaccines against infectious diseases. Acta Biomater..

[266] Scoles D.R., Minikel E.V., Pulst S.M. (2019). Antisense oligonucleotides: A primer. Neurol. Genet..

[267] Duharte A.B. Antisense Oligonucleotides for Vaccine Improvement. https://encyclopedia.pub/11540.

[268] Ying H., Ebrahimi M., Keivan M., Khoshnam S.E., Salahi S., Farzaneh M. (2021). miRNAs; a novel strategy for the treatment of COVID-19. Cell Biol. Int..

[269] Mehta A., Michler T., Merkel O.M. (2021). siRNA Therapeutics against Respiratory Viral Infections-What Have We Learned for Potential COVID-19 Therapies?. Adv. Health Mater..

[270] Karolinska Institutet Protective Gene Variant against COVID-19 Identified. https://www.sciencedaily.com/releases/2022/01/220113120742.htm.

[271] Huffman J.E., Butler-Laporte G., Khan A., Pairo-Castineira E., Drivas T.G., Peloso G.M., Nakanishi T., Ganna A., Verma A., Baillie J.K. (2022). Multi-ancestry fine mapping implicates OAS1 splicing in risk of severe COVID-19. Nature Genet..

[272] Pairo-Castineira E., Clohisey S., Klaric L., Bretherick A.D., Rawlik K., Pasko D., Walker S., Parkinson N., Fourman M.H., Russell C.D. (2021). Genetic mechanisms of critical illness in COVID-19. Nature.

[273] Niemi M.E., Karjalainen J., Liao R.G., Neale B.M., Daly M., Ganna A., Pathak G.A., Andrews S.J., Kanai M., Veerapen K. (2021). Mapping the human genetic architecture of COVID-19. Nature.

[274] Zeberg H., Pääbo S. (2021). A genomic region associated with protection against severe COVID-19 is inherited from Neandertals. Proc. Natl. Acad. Sci. USA.

[275] Bonnevie-Nielsen V., Field L.L., Lu S., Zheng D.-J., Li M., Martensen P.M., Nielsen T.B., Beck-Nielsen H., Lau Y.-L., Pociot F. (2005). Variation in antiviral 2′, 5′-oligoadenylate synthetase (2′ 5′ AS) enzyme activity is controlled by a single-nucleotide polymorphism at a splice-acceptor site in the OAS1 gene. Am. J. Hum. Genet..

[276] UK Research and Inovation Five Genes Identified That Could Be Key to New COVID-19 Treatments. https://www.ukri.org/news/five-genes-identified-that-could-be-key-to-new-covid-19-treatments/.

[277] Meara K. Genes May Be Key to New COVID-19 Treatments. https://www.contagionlive.com/view/genes-may-be-key-to-new-covid-19-treatments.

