# Supplementary material for: Nucleic Acid-Based COVID-19 Therapy Targeting Cytokine Storms: Strategies to Quell the Storm
Source: J Pers Med. 2022 Mar 3;12(3):386. doi: 10.3390/jpm12030386 (PMC8948998; doi:10.3390/jpm12030386)
Supplement: Supplementary file 1 [file jpm-12-00386-s001.zip › jpm-1594216-supplementary.pdf]

Journal of Personalized Medicine

MDPI

Table S1. List of RNA-based COVID-19 vaccine candidates.

Vaccine name(s)	Developer(s)	Stage of development	Components	Clinical trial no. / Reference(s)
Comirnaty/ BNT162	BioNTech & Pfizer	Authorized	Lipid nanoparticle-encapsulated mRNA encoding for SARS-CoV-2 S protein	ChiCTR2000034825, EudraCT 2020-001038-36, EudraCT 2020-003267-26, NCT04368728, NCT04380701, NCT04523571, NCT04537949, NCT04588480, NCT04649021, NCT04713553, NCT04754594; [36–51].
Spikevax/ mRNA-1273	Moderna Therapeutics	Authorized	Lipid nanoparticle-encapsulated mRNA encoding for SARS-CoV-2 S protein	NCT04283461, NCT04405076, NCT04470427, NCT04649151, NCT04677660, NCT04712110, NCT04847050; [52–60,70].
CVnCoV	CureVac AG	Phase III	Self-amplifying mRNA encoding for SARS-CoV-2 S protein	EudraCT 2020-004066-19, NCT04449276, NCT04515147, NCT04652102, NCT04674189, PER-054-20; ([61,62].
ARCoV	Walvax Biotechnology, Suzhou Abogen Biosciences, and the PLA Academy of Military Science	Phase II	Lipid nanoparticle-encapsulated mRNA encoding for the receptor-binding domain of SARS-CoV-2	ChiCTR2000034112, ChiCTR2000039212, ChiCTR2100041855, NCT04847102; [63].
HGCO19	Gennova Biopharmaceuticals	Phase I/II	Self-amplifying mRNA encoding for SARS-CoV-2 S protein	CTRI/2021/04/032688
EXG-5003	Elixirgen Therapeutics & Fujita Health University	Phase I/II	Small regulatory RNA	NCT04863131
Imperial's COVID-19 vaccine	Imperial College London/ VacEquity Global Health	Phase I/II	Self-amplifying mRNA encoding for SARS-CoV-2 S protein	ISRCTN17072692
ARCT-021	Arcturus Therapeutics	Phase I/II	Lipid nanoparticle-encapsulated mRNA encoding for SARS-CoV-2 S protein	NCT04480957, NCT04668339, NCT04728347
DS-5670	Daiichi-Sankyo Institute	Phase I/II	mRNA	NCT04821674
PTX-COVID	Providence	Phase I	Lipid nanoparti-	NCT04765436; [64,65].

19-B	Therapeutics Holdings		cle-encapsulated mRNA encoding for SARS-CoV-2 S protein	

NCT, ISRCTN, ChiCTR CTRI, and EudraCT numbers refer to trials registered in ClinicalTrials.gov, ISRCTN Registry, Chinese Clinical Trial Registry, Clinical Trials Registry - India, and European Union Drug Regulating Authorities Clinical Trials Databases, respectively.

Table S2. List of RNA-based treatments against COVID-19.

Developer(s)	Stage of development	Product	Clinical trial no. / Reference(s)
Mateon Therapeutics	Clinical - Phase II study IND submitted to FDA on April 27, 2020; Phase II trials approved in Peru Nov 2020	OT-101, a TGF-Beta antisense drug candidate	[87]
AIM ImmunoTech/ National Institute of Infectious Diseases in Japan/ Roswell Park Comprehensive Cancer Center	Clinical - Phase I/II trial of Ampligen (rintatolimod), in combination with interferon alfa-2b, in cancer patients with COVID-19 not yet recruiting July 2020	Ampligen; (rintatolimod)	NCT04379518; [88,89]
Irnaomics	Pre-clinical	RNAi - testing 150 RNAis	[90]
Vir Biotech / Alnylam Pharmaceuticals	Pre-clinical	VIR-2703 (ALN-COV) siRNA candidate	-
Neurimmune/ Ethris	Pre-clinical	Inhaled mRNA	-
Sarepta Therapeutics / US Army Medical Research Institute of Infectious Diseases (USAMRIID)	Pre-clinical	Antisense oligonucleotides, peptide conjugated	-

NCT number refers to a trial registered in ClinicalTrials.gov

Table S3. DNA-based vaccine candidates against COVID-19.

Vaccine name(s)	Developer(s)	Stage of development	Components	Clinical trial no. / Reference(s)
ZyCoV-D	Zydus Cadila Healthcare	Clinical - Phase III	DNA plasmid vaccine	CTRI/2020/07/026352, CTRI/2021/01/030416
AG0302/ AG0301-COVID	AnGes Inc. & Osaka University	Clinical - Phase II/III	AG0301 & AG0302 plasmid vaccine with adjuvant	jRCT2051200085, jRCT2051200088, NCT04463472, NCT04527081, NCT04655625
INO-4800	Inovio Pharmaceuticals	Clinical - Phase II/III	DNA plasmid vaccine with electroporation	ChiCTR2000038152, ChiCTR2000040146, NCT04336410, NCT04447781,

				NCT04642638; [105,108–110]
GX-19N	Genexine & GenNBio	Clinical - Phase I/II		NCT04445389, NCT04715997
GLS-5310	GeneOne Life Science	Clinical - Phase I/II		NCT04673149
COVID-eVax	Takis Biotech	Clinical - Phase I/II		EudraCT2020-003734-20, NCT04788459
Covigenix	Entos Pharmaceuticals	Clinical - Phase I/II began April 2021		NCT04591184
CORVax12	OncoSec Medical Incorporated	Clinical - Phase I	Interleukin-12 expression platform with S glycoprotein	NCT04627675
CoVAXIX	Statens Serum Institute	Clinical - Phase I began early 2021	DNA plasmid vaccine	[106]
bacTRL-Spike	Symvivo	Clinical - Phase I began November 2020		NCT04334980
COVIGEN	BioNet Asia	Clinical - Phase I began end of June 2021	Needle-free delivery	NCT04742842
DIOS-CoVax2	University of Cambridge & DIOSynVax	Clinical - Phase I began early 2021	Synthetic gene inserts compatible with multiple delivery systems	[107]
msDNA-VLP	Mediphage Bioceuticals	Pre-clinical		-
-	Immunomic Therapeutics & EpiVax	Pre-clinical	DNA plasmid vaccine, needle-free delivery	-
-	OPENCORONA, a project by Karolinska Institute and collaborators	Pre-clinical	DNA with electroporation	-
-	Chula Vaccine Research Center	Pre-clinical	DNA with electroporation	-
-	National Research Center, Egypt	Pre-clinical	DNA plasmid vaccine S, S1, S2, RBD and N	-
-	Ege University Drug Development and Pharmacokinetic Research Application Center (ARGEFAR)	Pre-clinical	DNA	-
-	Globe Biotech	Pre-clinical	DNA plasmid vac-	-

	Limited, Bangla-desh		cine	
-	National Institute of Chemistry, Slovenia	Pre-clinical	DNA plasmid, nanostructured RBD	-

NCT, ChiCTR CTRI, jRCT, and EudraCT numbers refer to trials registered in ClinicalTrials.gov, Chinese Clinical Trial Registry, Clinical Trials Registry - India, Japan Registry for Clinical Trials, and European Union Drug Regulating Authorities Clinical Trials Databases, respectively.
